# Synthesis of RNA-cofactor conjugates and structural exploration of RNA recognition by an m^6^A RNA methyltransferase

**DOI:** 10.1093/nar/gkac354

**Published:** 2022-05-17

**Authors:** Vincent Meynier, Laura Iannazzo, Marjorie Catala, Stephanie Oerum, Emmanuelle Braud, Colette Atdjian, Pierre Barraud, Matthieu Fonvielle, Carine Tisné, Mélanie Ethève-Quelquejeu

**Affiliations:** Expression Génétique Microbienne, UMR 8261, CNRS, Université Paris Cité, Institut de Biologie Physico-Chimique (IBPC), 75005, Paris, France; Laboratoire de Chimie et Biochimie Pharmacologiques et Toxicologiques, UMR 8601, CNRS, Université Paris Cité, 75006, Paris, France; Expression Génétique Microbienne, UMR 8261, CNRS, Université Paris Cité, Institut de Biologie Physico-Chimique (IBPC), 75005, Paris, France; Expression Génétique Microbienne, UMR 8261, CNRS, Université Paris Cité, Institut de Biologie Physico-Chimique (IBPC), 75005, Paris, France; Laboratoire de Chimie et Biochimie Pharmacologiques et Toxicologiques, UMR 8601, CNRS, Université Paris Cité, 75006, Paris, France; Laboratoire de Chimie et Biochimie Pharmacologiques et Toxicologiques, UMR 8601, CNRS, Université Paris Cité, 75006, Paris, France; Expression Génétique Microbienne, UMR 8261, CNRS, Université Paris Cité, Institut de Biologie Physico-Chimique (IBPC), 75005, Paris, France; Sorbonne Université, Université Paris Cité, Centre de recherche des Cordeliers, 75006, Paris, France; Expression Génétique Microbienne, UMR 8261, CNRS, Université Paris Cité, Institut de Biologie Physico-Chimique (IBPC), 75005, Paris, France; Laboratoire de Chimie et Biochimie Pharmacologiques et Toxicologiques, UMR 8601, CNRS, Université Paris Cité, 75006, Paris, France

## Abstract

Chemical synthesis of RNA conjugates has opened new strategies to study enzymatic mechanisms in RNA biology. To gain insights into poorly understood RNA nucleotide methylation processes, we developed a new method to synthesize RNA-conjugates for the study of RNA recognition and methyl-transfer mechanisms of SAM-dependent m^6^A RNA methyltransferases. These RNA conjugates contain a SAM cofactor analogue connected at the N6-atom of an adenosine within dinucleotides, a trinucleotide or a 13mer RNA. Our chemical route is chemo- and regio-selective and allows flexible modification of the RNA length and sequence. These compounds were used in crystallization assays with RlmJ, a bacterial m^6^A rRNA methyltransferase. Two crystal structures of RlmJ in complex with RNA–SAM conjugates were solved and revealed the RNA-specific recognition elements used by RlmJ to clamp the RNA substrate in its active site. From these structures, a model of a trinucleotide bound in the RlmJ active site could be built and validated by methyltransferase assays on RlmJ mutants. The methyl transfer by RlmJ could also be deduced. This study therefore shows that RNA-cofactor conjugates are potent molecular tools to explore the active site of RNA modification enzymes.

## INTRODUCTION

**Figure 1.**
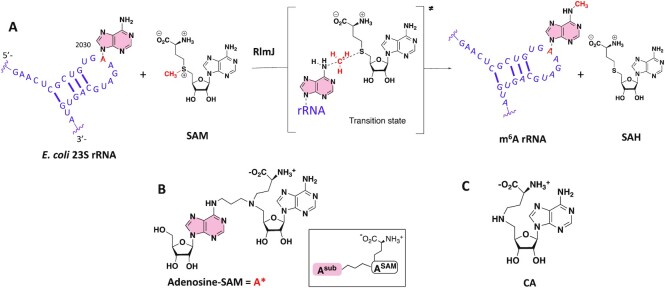
(**A**) RlmJ-catalyzed transfer of the methyl group of SAM to N6-atom of A2030 of the *E. coli* 23S rRNA. The minimal RNA substrate for RlmJ is drawn in purple. (**B**) Bisubstrate analogue (A*) previously described (14,15). The inset shows the abbreviations used to differentiate the two adenosines contained in the adenosine-SAM conjugates. (**C**) SAM cofactor analogue (**CA**).

RNAs are heavily co- or post-transcriptionally modified. To date, 152 chemical modifications of bases and ribose have been described, with the largest diversity and highest number of modifications found in transfer RNA (tRNA) (1–4). The mono-methylation of the exocyclic amine of adenine (m^6^A) is found in all organisms from bacteria to human and in mRNA, tRNA, rRNA, small nucleolar RNA, long non-coding RNA and microRNA. It is the most commonly occurring internal modification in mRNA from eukaryotes. m^6^A is reversible on mRNA, meaning that distinct sets of proteins introduce (m^6^A RNA methyltransferases called the writers), recognize (the readers) and remove (the erasers) this epitranscriptomic mark, thus allowing additional levels of regulation of mRNA translation. It has yet to be conclusively determined if such a writer/reader/eraser system exists for m^6^A on other types of RNA. The regulatory mechanism of the m^6^A modification is complex (5,6) and its dysregulation is linked to human diseases (7–9) and virus replication (10–12).

m^6^A RNA methyltransferases (MTases) catalyze the transfer of the methyl group from the cofactor S-adenosyl-L-methionine (SAM, Figure 1A) to the N6-atom of adenines at specific positions in RNA, releasing the cofactor product *S*-adenosyl-l-homocysteine (SAH, Figure 1A). Six m^6^A RNA MTases have been structurally characterized so far (3,4), but only one enzyme, METTL16, has been crystallized in complex with a substrate RNA (PDB: 6DU4 and 6DU5) (13). Although the methyltransferase domains of the six MTases superimpose well, the structure of the METTL16/RNA complex does not allow an understanding of RNA recognition by the other m^6^A RNA MTases. In fact, superimposition of RNA-bound METTL16 with other structurally characterized m^6^A MTases reveals steric clashes between the protein and the RNA for all enzymes, indicating that their specific RNA-substrate recognition differs from that of METTL16. The limited number of RNA-bound m^6^A MTase structures reflects the difficulty to solve the structure of modification enzymes in complex with RNA. Consequently, the RNA recognition and methylation reaction mechanism of m^6^A RNA MTases are still poorly understood. In contrast, structures are available for most MTases in complex with either the SAM cofactor or the cofactor product, SAH, allowing for a well-understood cofactor binding pattern within this family of proteins. The cofactor binding guided the initial design of bisubstrate analogues as chemical tools to mimic the state at which both the substrate nucleoside and the SAM cofactor are bound in the catalytic pocket of the enzyme (14–17). In recent years, there have been significant efforts made toward the development of SAM analogues with the aim to study the transfer of moieties other than simple methyl groups (18,19), to obtain protein inhibition (20) or to study SAM-dependent DNA-methyltransferase mechanisms (21,22). Here, the aim is to facilitate protein/RNA crystallization to gain insights into currently poorly understood RNA nucleotide methylation processes.

**Figure 2.**
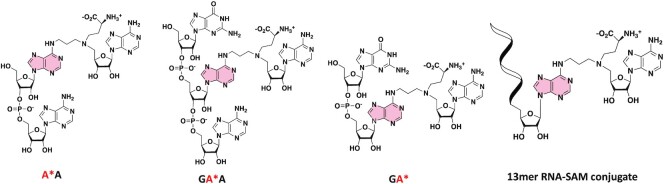
RNA–SAM conjugates synthesized in this work to explore the RNA recognition by RlmJ.

We previously designed adenosine-SAM bisubstrate analogues that contain the SAM cofactor with the S-atom replaced by a N-atom directly linked to an adenosine at the point of nucleophilic attack to mimic the substrate/cofactor bound state (14–16) (Figure 1B). These molecules were used to study the substrate recognition of the m^6^A MTase RlmJ, the Ribosomal RNA large subunit methyltransferase J that N6-methylates the adenine A2030 of the *E. coli* 23S rRNA (23) (Figure 1A). RlmJ is a good example of an m^6^A MTase that was successfully crystallized with SAM (PDB 4BLV), SAH (PDB 6QE5) and SAM plus AMP as the adenine substrate analogue (PDB 4BLW), but not with an RNA substrate (23,24). In the AMP-bound RlmJ structure, the exocyclic N6-atom of the AMP-adenine was outside hydrogen-bonding distance from potential proton acceptors and was unlikely to mimic the true adenosine substrate (A^sub^) position. In contrast, we previously showed that the bisubstrate analogue A* (Figure 1B), containing a SAM cofactor analogue **CA** (Figure 1C) tethered to A^sub^ at the N6-atom by an alkyl linker of three carbons, crystallized in the active site of RlmJ (15). The adenine is positioned in the presumed substrate binding pocket of RlmJ. For the cofactor moiety, the methionine chain is bound like SAM but the adenosine of the SAM analogue (A^SAM^) is rotated out of the canonical binding pocket for SAM. In the present study, we show that **CA** is able to bind correctly in the active site of RlmJ. CA is therefore used as a universal building block and we develop a new chemical strategy to increase the size of the RNA tethered to CA (Figure 2).

**Scheme 1.**
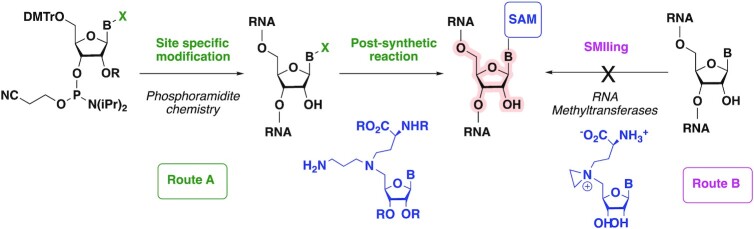
Routes for the synthesis of RNA-cofactor conjugates.

These RNA-cofactor conjugates could be synthetized using two different strategies: (i) a post-synthetic approach, which involves the introduction of a reactive group into the RNA followed by a reaction with the cofactor in a selective manner (Scheme 1, route A) or (ii) a chemo-enzymatic approach called SMILing for ‘Sequence-specific Methyltransferase-Induced Labeling’ (Scheme 1, route B) based on alkylation reactions catalysed by a MTase in presence of cofactor analogues (25,26). This SMILing method has been previously described for modification of proteins (27) and DNA (26,28). In this approach, the nucleophilic attack of the substrate adenosine is expected to open the putative aziridine ring of the SAM analogue and directly provide an adenosine-SAM conjugate tethered by a two-carbon linker. The synthesis of RNA–SAM conjugates with three-carbon linker therefore requires the development of a post-synthetic approach (Scheme 1, route A) with a chemo-selective post-functionalization step to connect the SAM cofactor analogue to the N6-atom of a specific adenosine in the RNA.

Here, we show that nucleophilic aromatic substitution as a post-synthetic reaction can be efficiently used to synthesize four RNA–SAM conjugates, in which the **CA** SAM analogue is connected to the N6-atom of an adenosine. This modified adenosine is located at the 5′-end or 3′-end of a dinucleotide, in the internal position of a trinucleotide or within a 13-mer RNA with the sequence of the RNA substrate of RlmJ (Figure 2). These compounds were then used in crystallization assays in complex with RlmJ. Two crystal structures were solved with an RNA–SAM conjugate bound in the active site of RlmJ. These structures reveal the specific recognition mode of RNA by RlmJ, which was further confirmed by mutation and MTase activity studies and decipher the mechanism of methyl transfer used by RlmJ.

## MATERIALS AND METHODS

### General information

Reactions were carried out under argon atmosphere and performed using freshly distilled solvents. Dimethyl formamide (DMF) and MeOH were dried over calcium hydride. Phosphoramidite nucleosides, tetrazole and solvents for dinucleotides synthesis were purchased from Eurogentec. Progress of the reactions was monitored by thin layer chromatography (TLC). TLC: precoated silica gel thin layer sheets 60 F_254_ (Merck, Darmstadt, Germany) and detection by charring with 10% H_2_SO_4_ in ethanol followed by heating.

Preparative HPLC were performed using a Reverse-phase HPLC system (Shimadzu, Marne-la-Vallée, France) with a reverse phase C-18 NUCLEOSIL column (250 mm × 21.2 mm, 5 μm) using a solvent system consisting of A: 50 mM aqueous NH_4_OAc pH 4.5 and B: MeCN (linear gradient from 0% B to 63% B in 30 min) at a flow rate of 15 ml/min and UV detection at 254 nm.

The NMR characterizations for all synthesized compounds are presented in the Supplementary data and the NMR spectra are provided in Supplementary Figures S1–S29. The reverse-phase HPLC chromatograms of the final compounds (A*A, GA*A, GA* and the **13mer RNA–SAM conjugate**) are shown in Supplementary Figures S30–S36.

### Chemical synthesis of compound 7 (Scheme 2)

Inosine (2.0 g, 7.45 mmol) was dissolved in DMF (20 ml) and di-*tert*-butylsilyl bis(trifluoromethanesulfonate) (2.67 ml, 8.19 mmol) was added dropwise at 0°C. The reaction was stirred at 0°C for 30 min and imidazole (2.53 g, 37.25 mmol) was added. The reaction was allowed to warm to room temperature (rt) and stirred for an additional hour. *tert*-butyl chloride (2.24 g, 14.9 mmol) and imidazole (1.01 g, 14.9 mmol) were added successively at 0°C and the reaction was stirred at room temperature overnight. The reaction was diluted with ethyl acetate (EtOAc) and washed five times with brine. The combined organic layers were dried over anhydrous MgSO_4_, filtered, and concentrated. The residue was purified by silica gel chromatography (eluent: cyclohexane/EtOAc 5:5) to provide the compound **1** as a white foam (3.9 g, quantitative yield). To a solution of **1** (3.45 g, 6.6 mmol) in THF (60 ml), hydrogen fluoride pyridine complex (1.48 ml, 16.5 mmol) in pyridine (2.5 ml) was added at 0°C and the mixture was stirred at room temperature for 15 min. The reaction was diluted with Dichloromethane (DCM) and extracted with sat. NaHCO_3_. The organic layer was dried over anhydrous MgSO_4_, filtered, and concentrated under reduced pressure. The crude residue was purified by silica gel chromatography using DCM/MeOH (9/1) as the eluent to afford compound **2** as a white foam (1.74 g, 69%). Compound **2** (1.74 g, 4.5 mmol) was dissolved in pyridine (7 ml). 4,4′-Dimethoxytrityl chloride (1.69 g, 4.9 mmol) was added at 0°C and the reaction was stirred at 0°C for 16 h. The reaction was diluted with DCM, washed with brine, dried over anhydrous MgSO_4_, filtered, and concentrated. The residue was purified by silica gel chromatography (eluent: DCM/MeOH 96:4) to provide the desired compound **3** as a white foam (1.01 g, 32%). Benzotriazol-1-yloxytripyrrolidinophosphonium hexafluorophosphate (PyBOP) (689 mg, 1.55 mmol) and diisopropylethylamine (DIPEA) (339 μl, 1.93 mmol) were added at 0°C to a solution of compound **3** (888 mg, 1.29 mmol) in DMF (5 ml) and the reaction mixture was stirred at room temperature for 16 h. The mixture was partitioned between EtOAc and brine. The organic layer was dried over anhydrous MgSO_4_, filtered and concentrated. The residue was purified by silica gel chromatography (cyclohexane/EtOAc 7:3) to provide the compound **4** (384 mg, 37%). Compound **4** (464 mg, 0.57 mmol) was dissolved in DCM (7 ml). 2-Cyanoethyl *N*,*N*-diisopropylchlorophosphoramidite (516 μl, 2.28 mmol) and DIPEA (403 μl, 2.28 mmol) were added to the reaction mixture at 0°C. The solution was stirred at room temperature for 16 h, diluted with DCM and washed with a saturated solution of NaHCO_3_ and brine. The organic layer was dried over anhydrous MgSO_4_, filtered, and concentrated. The reaction was purified by silica gel chromatography neutralized with DIPEA (eluent: cyclohexane/EtOAc 7:3) to provide the two diastereoisomers **5** as a white foam (424 mg, 73%). To a solution of phosphoramidite **5** (184 mg, 180 μmol) in MeCN (2 ml) was added tetrabenzoyl-adenosine **6** (70 mg, 100 μmol). The reaction mixture was stirred at room temperature for 30 min and a 0.45 M tetrazole solution in MeCN (2.2 ml, 1 mmol) was added. After stirring at room temperature for 20 h, a 0.1 M iodine solution in THF/H_2_O/Pyridine (75/2/20, 3.1 ml) was added. After 1 h, the reaction mixture was diluted with EtOAc, washed with water, a saturated solution of Na_2_S_2_O_3_ and brine. The organic layers were combined, dried over anhydrous MgSO_4,_ filtered, and concentrated under vacuo. The residue was then stirred with a 0.18 M trichloroacetic acid (TCA) solution in DCM (5.7 ml) at room temperature for 30 min. The reaction mixture was diluted with DCM and the organic layer washed with a saturated solution of NaHCO_3_ and brine, dried over anhydrous MgSO_4_, filtered, and evaporated. The residue was purified by silica gel chromatography using DCM/MeOH (98/2) as eluent to afford compound **7** as a white foam (50 mg, 38% over three steps).

### **Chemical synthesis of compound 10** (Scheme 3)

Compound **7** (183 mg, 140 μmol) and Ac-G-PCNE phosphoramidite (250 mg, 266 μmol) were stirred at room temperature for 30 min in MeCN (2 ml). A 0.45 M tetrazole solution in MeCN (3.1 ml, 1.4 mmol) was added and the reaction mixture was stirred at room temperature. After 20 h, a 0.1 M iodine solution in THF/H_2_O/Pyridine (75/2/20, 4.2 ml) was added and stirred for 1 h. The reaction mixture was then diluted with EtOAc, washed with water, a saturated solution of Na_2_S_2_O_3_ and brine. The organic layers were combined, dried over anhydrous MgSO_4,_ filtered, and concentrated under vacuo. The residue was then stirred with a 0.18 M TCA solution in DCM (7.8 ml) at room temperature for 30 min. The reaction mixture was diluted with DCM and the organic layer washed with a saturated solution of NaHCO_3_ and brine, dried over anhydrous MgSO_4_, filtered, and evaporated. The residue was purified by silica gel chromatography using DCM/MeOH (96/4) as eluent to afford compound **10** as a white foam (129 mg, 49% over three steps).

### Chemical synthesis of compound 15 (Scheme 4)

To a solution of inosine (1.0 g, 3.7 mmol, 1 eq) and imidazole (2.03 g, 29.8 mmol, 8 eq) in DMF (11 ml) was added *tert*-butyldimethylsilyl chloride (TBDMSCl) (2.25 g, 14.9 mmol, 4 eq) at 0°C. The reaction mixture was then stirred at 50°C for 16 h. The residue was partitioned between EtOAc and brine. The organic phase was washed with brine, dried over anhydrous MgSO_4_, filtered, and concentrated. The residue was purified by silica gel chromatography (cyclohexane/EtOAc 5:5) to provide the compound **12** as a white foam (1.96 g, 87%). PyBOP (618 mg, 1.40 mmol) and DIPEA (304 μl, 1.74 mmol) were added at 0°C to a solution of inosine **12** (711 mg, 1.16 mmol) in DMF (5 ml) and the reaction mixture was stirred at room temperature for 16 h. The residue was partitioned between EtOAc and brine. The organic layer was dried over anhydrous MgSO_4_, filtered, and concentrated. The residue was purified by silica gel chromatography (cyclohexane/EtOAc 9:1) to provide the compound **13** as a white foam (677 mg, 80%). A 1/1 (v/v) mixture of TFA/H_2_O (3.5 ml, 46 mmol) was added at 0°C to a solution of compound **13** (677 mg, 0.92 mmol) in THF (20 ml). The reaction mixture was stirred at room temperature for 1h30. EtOAc and a saturated solution of NaHCO_3_ were added and the organic phase was washed with brine. The organic layer was dried over anhydrous MgSO_4_, filtered and concentrated. The residue was purified by silica gel chromatography using cyclohexane/EtOAc (7/3) as eluent to afford compound **14** (361 mg, 63%) as a white foam. Compound **14** (85 mg, 138 μmol) in MeCN (2 ml) was added to a solution of Ac-G-PCNE phosphoramidite (250 mg, 262 μmol) in MeCN (300 μl). The reaction mixture was stirred at room temperature for 30 min and a 0.45 M tetrazole solution in MeCN (3 ml, 1.38 mmol) was added. After stirring at room temperature for 20 h, a 0.1 M iodine solution in THF/H_2_O/Pyridine (75/2/20, 4.1 ml) was added. After 1 h, the reaction mixture was diluted with EtOAc, washed with water, a saturated solution of Na_2_S_2_O_3_ and brine. The organic layers were combined, dried over anhydrous MgSO_4,_ filtered, and concentrated under vacuo. The residue was then stirred with a 0.18 M TCA solution in DCM (7.6 ml) at room temperature for 30 min. The reaction mixture was diluted with DCM and the organic layer washed with a saturated solution of NaHCO_3_ and brine, dried over anhydrous MgSO_4_, filtered, and evaporated. The residue was purified by silica gel chromatography using DCM/MeOH (96/4) as eluent to afford compound **15** as a white foam (91 mg, 56% over three steps).

### Chemical synthesis of compound 18 (Scheme 5)

To a solution of iPr-Pac-dG-PCNE phosphoramidite (500 mg, 0.53 μmol) in MeCN (600 μl) was added compound **14** (111 mg, 0.18 mmol) in MeCN (4 ml). The reaction mixture was stirred at room temperature for 30 min and a 0.45 M tetrazole solution in MeCN (4 ml, 1.8 mmol) was added. After stirring at room temperature for 20 h, a 0.1 M iodine solution in THF/H_2_O/Pyridine (75/2/20, 5.4 ml) was added. After 1 h, the reaction mixture was diluted with EtOAc, washed with water, a saturated solution of Na_2_S_2_O_3_ and brine. The organic layers were combined, dried over anhydrous MgSO_4,_ filtered, and concentrated under vacuo. The residue was then stirred with a 0.18 M TCA solution in DCM (10 ml) at room temperature for 30 min. The reaction mixture was diluted with DCM and the organic layer washed with a saturated solution of NaHCO_3_ and brine, dried over anhydrous MgSO_4_, filtered, and evaporated. The residue was purified by silica gel chromatography using DCM/MeOH (96/4) as eluent to afford compound **17** (147 mg, 69% over three steps). Compound **17** (147 mg, 0.12 mmol) was dissolved in MeCN (1 ml) and bis(2-cyanoethyl)diisopropylphosphoramidite (102 mg, 0.36 mmol) in MeCN (1 ml), followed by tetrazole (2.8 ml, 1.2 mmol, 0.45 M solution in MeCN) were added. The mixture was stirred at room temperature for 3 h and a 0.1 M solution of I_2_, in THF/H_2_O/Pyridine (75/2/20, 3.8 ml) was added. After being stirred at room temperature for 1 h, the mixture was diluted with EtOAc and washed successively with a saturated solution of Na_2_S_2_O_3_ and brine, dried over anhydrous MgSO_4,_ filtered, and concentrated to dryness. The residue was purified by silica gel chromatography using DCM/MeOH (9/1) as eluent to afford compound **18** (134 mg, 85% over 2 steps).

### Chemical synthesis of A*A, GA*A and GA* (Schemes 3, 4 and 5)

SAM analogue **8** (1,2 equivalent) and DIPEA (12 equivalent) were added at 0°C to a solution of compound **7**, **10**, **15** or **18** (1 equivalent) in DMF and the reaction mixture was stirred at room temperature for 24 h. After concentration, the residue was dissolved in a 5 M solution of MeNH_2_ (EtOH/H_2_O, 1/1) (200 equivalent) and the reaction was stirred at room temperature for 24 h and concentrated. The residue was then dissolved in MeOH and CsF (200 equivalent) was added. The reaction mixture was stirred at 60°C for 24 h. After concentration under vacuo, the residue was purified using a Reverse-phase HPLC system. The appropriate fractions were collected and lyophilized, to give protected intermediates **9**, **11**, **16** or **19**, respectively. Compound **9**, **11**, **16** or **19** (1 equivalent) was dissolved in a 5 M solution of ZnBr_2_ in a 1/1 (v/v) mixture of iPrOH/MeNO_2_ (400 equivalent) and the reaction mixture was stirred at room temperature for 24 h. Water was added, and the mixture was lyophilized. The residue was then purified using a Reverse-phase HPLC system. The appropriate fractions were collected and lyophilized to give compound A*A, GA*A or GA*.

### Synthesis of the 13mer RNA–SAM conjugate (Scheme 5)

SAM analogue **8** (160 mg, 0.18 mmol) and DIPEA (355 μl, 2.04 mmol) were added at 0°C to a solution of compound **18** (231 mg, 0.17 mmol) in DMF (3 ml) and the reaction mixture was stirred at room temperature for 24 h. After concentration, the residue was dissolved in a 5 M solution of MeNH_2_ (EtOH/H_2_O, 1/1) (6.8 ml) and the reaction was stirred at room temperature for 24 h and concentrated. The residue was then dissolved in MeOH (15 ml) and CsF (5.16 g, 34 mmol) was added. The reaction mixture was stirred at 60°C for 24 h. After concentration under vacuo, the residue was purified using a Reverse-phase HPLC system. The appropriate fractions were collected and lyophilized to give the protected dinucleotide **19** (14 mg, 7% over 3 steps). Dinucleotide **19** (14 mg, 11 μmol) was dissolved in a 5 M solution of ZnBr_2_ in a 1/1 (v/v) mixture of iPrOH/MeNO_2_ (452 μl, 2.2 mmol) and the reaction mixture was stirred at room temperature for 24 h. Water was added, and the mixture was lyophilized. The residue was purified using a Reverse-phase HPLC system. The appropriate fractions were collected and lyophilized to give the dinucleotide pdGA* (0.4 mg, 4%).

#### Synthesis of **11mer RNA** (5′-GAACUCGCUGU-3′)

The 11mer RNA sequence (5′-GAACUCGCUGU-3′) was synthesized automatically (1 μmol scale) with the use of an H-2 GeneWorld DNA/RNA automated synthesizer (K&A, Laborgeraete GbR, Schaafheim, Germany). More information is given in the Supplementary data.

#### Enzymatic ligation

The ligation of pdGA* (200 nmol) to the 11-mer RNA (20 nmol) was performed with purified T4 RNA ligase, in the presence of DMSO (10%), ATP (1 mM) and MgCl_2_ (15 mM) in 500 μl of 50 mM HEPES buffer pH 7.5 at 30°C for 12 h (Supplementary data, page 23). The 13mer RNA–SAM conjugate was characterized by mass spectrometry and analyzed by reverse-phase HPLC.

### Cloning, expression and purification of RlmJ and mutants

RlmJ (residues 1–280) was subcloned into the pET15b vector bearing an N-terminal His6-tag followed by a thrombin protease cleavage site as previously described (15). Mutations were generated using the QuikChange Site-Directed Mutagenesis Kit (Agilent), and verified by sequencing. Plasmids were transformed into *E. coli* BL21(DE3) competent cells, cultured in Lysogeny Broth at 37°C, and protein expression was induced with 0.5 mM isopropyl β-d-1-thiogalactopyranoside for 3 h at 37°C. Cell pellets were lysed by sonication in a buffer containing 20 mM Tris–HCl pH 7.0, 200 mM NaCl, 5% (v/v) glycerol and 5 mM β-mercaptoethanol, clarified by centrifugation. The clarified lysate was applied to a 5 ml HisTrap HP column (Cytiva) and eluted with a gradient of 20–500 mM imidazole. RlmJ was treated with thrombin protease for 4 h at 22°C to remove the His6-tag and applied to a 5 ml HisTrap HP column (Cytiva) to trap the His6-tag. Further purification of RlmJ was performed by size exclusion chromatography (SEC) on Superdex S75 PG (Cytiva). RlmJ was concentrated and stored at –80°C in the SEC buffer (20 mM Tris–HCl pH 7.0, 150 mM NaCl, 5% (v/v) glycerol and 5 mM β-mercaptoethanol).

### Crystallization, data collection and structure determination

Crystallization assays were conducted at 18°C using the sitting-drop vapor-diffusion method and the Hampton Research Index HT™ screen, for which crystals of RlmJ in complex with SAM or SAH are routinely produced. RNA–SAM conjugates were dissolved in H_2_O at 10 mM final concentration, except for the 13mer RNA–SAM that was dissolved at 1 mM final concentration. RlmJ and RNA–SAM conjugates were mixed at ratios ranging from 1/2 to 1/10 before crystallization assays with RlmJ concentrated at around 10 mg/ml. For **CA**, A*A and GA*, all the conditions of the screen were performed at different RlmJ/RNA–SAM ratios. For GA*A, only five lines (D to H) of the Hampton Research Index HT^TM^ screen were tested with a RlmJ/GA*A ratio of 1/2, because the amount of GA*A was limiting (217 nmol). Three conditions, where crystals grew without ligand bound to RlmJ when using the 1/2 ratio, were performed again by increasing the RlmJ/GA*A ratio to 1/8. For the 13mer RNA–SAM (3.7 nmol available for crystallogenesis assays), only two lines (D and H) at a RlmJ/13mer RNA–SAM ratio of 1/2 could be tested with a final protein concentration of 5.3 mg/ml.

RlmJ bound to **CA** (RlmJ/**CA** ratio of 1/10) crystallized with a final protein concentration in the drop of 4.75 mg/ml, in a 150 nL drop with a 40 μl reservoir solution containing 0.1 M Bis–Tris pH 6.5, 28% (w/v) PEG MME 2000. RlmJ bound to GA* (RlmJ/GA* ratio of 1/2) crystallized with a final protein concentration of 5.5 mg/ml, in a 400 nL drop with a 40 μl reservoir solution containing 0.1 M Bis–Tris pH 6.5, 0.2 M NaCl, 25% (w/v) PEG 3350. RlmJ bound to GA*A (RlmJ/GA*A ratio of 1/8) crystallized with a final protein concentration of 5.0 mg/ml, in a 200 nL drop (100 + 100) with a 20 μl reservoir solution containing 0.1 M Bis–Tris pH 6.5, 20% (w/v) PEG MME 5000.

Crystals were cryoprotected with reservoir solution supplemented with 19, 23 or 21% (v/v) glycerol for crystals with **CA**, GA* or GA*A respectively, and flash frozen in liquid nitrogen. Diffraction data were collected at the Synchrotron Soleil beamline PX1 or PX2 and refined to 2.09 Å for RlmJ/**CA**, 2.29 Å for RlmJ/GA* and 1.59 Å for RlmJ/GA*A. Diffraction data were processed using XDS (29). Phases were determined by molecular replacement in PHASER (30) in the CCP4 Suite of programs (31), using an existing RlmJ structure as search model (PDB 6QDX). Modelling and refinement were carried out using Refmac (32), COOT (33) and Phenix (34). Density was visible for all residues aside from loopy parts covering residues 53–57 in RlmJ/**CA**, residues 54–58 of RlmJ/GA* and residues 52–57 in chain A and residues 52–56 in chain B of RlmJ/GA*A.

### Differential scanning fluorimetry (DSF)

DSF was performed in a 96-well plate using a CFX96 Touch real-time PCR detection system (Bio-Rad) with excitation and emission filters of 450–490 and 515–530 nm, respectively. Each 20 μl well reaction was carried out in the DSF buffer (20 mM Tris–HCl pH 8.0, 150 mM NaCl, 5% (v/v) glycerol and 5 mM β-mercaptoethanol) and consisted of 2 μl protein at a final concentration of 5 μM, 2 μl SYPRO ORANGE diluted 500-fold from the manufacturer's stock (i.e. 5000× in DMSO (Invitrogen)) in the SEC buffer and (if applicable) 2 μl *S*-adenosyl-l-methionine (SAM) (Merck) to a final concentration of 1 mM. Fluorescence intensities were measured from 25 to 85°C with a ramp rate of 1°C/min. The melting temperature *T*_m_ was determined by curve-fitting using GraphPad Prism v.7.0 software (35).

### Methyltransferase activity assays

(GG)H72 RNA (27 nucleotides, sequence 5′-GGGAACUCGCUGUGAAGAUGCAGUGUA-3′) was purchased deprotected and desalted from Dharmacon (HorizonTM). It was resuspended in the activity buffer (20 mM potassium phosphate pH 6.5, 50 mM NaCl, 5 mM β-mercaptoethanol) to a 500 μM concentration, heated for 2 min at 95°C then cooled down on ice for at least 15 min. A reaction contained 2000 pmol (GG)H72 RNA supplemented with 3000 pmol unlabeled SAM (Sigma-Aldrich) doped with 5 pmol *S*-adenosyl-l-[methyl-3*H*]-methionine (80 Ci/mmol, American Radiolabeled Chemicals, Inc.) and followed by the addition of 200 pmol wild-type or mutant RlmJ or buffer alone. All reactions were carried out at 37°C for 1h in a 50 μl final volume of the activity buffer previously described, then quenched in 1.5 ml of ice cold 15% (w/v) trichloroacetic acid and incubated on ice for at least 10 min. The precipitates were collected by filtration using GF/C filters (Whatman) under vacuum and washed four times with 5 ml of cold 15% (w/v) trichloroacetic acid. The washed and dried filters were then placed in vials containing 5 ml of liquid scintillation cocktail (PerkinElmer), shaken for 15 min and counted in an Hidex 300 SL scintillation counter (LabLogic ScienceTec). All assays were done within 1 day of enzyme purifications, as the enzyme is unstable when stored over time.

## RESULTS AND DISCUSSION

### Chemical synthesis of RNA–SAM conjugates

The synthesis of RNA–SAM conjugates was achieved by a chemo-selective nucleophilic aromatic substitution (S_N_Ar) step to connect CA via a 3-carbon alkyl chain to the N6-atom of a specific adenosine of the RNA. Here, the convertible nucleoside was introduced at the 5′-end or in an internal position of the RNA using the O^6^-(benzotriazolyl)inosine phosphoramidite **5** and at the 3′-end of the RNA using the O^6^-(benzotriazolyl)inosine **14** by phosphoramidite chemistry.

### Synthesis of the di- and tri- nucleotides–SAM conjugates

**Scheme 2.**
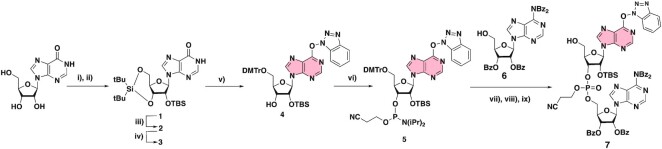
Synthesis of compound **7** from inosine. (i) di-*tert*-butylsilyl bis(trifluoromethanesulfonate), imidazole, DMF, rt, 1 h 30, (ii) TBDMSCl, imidazole, DMF, rt, 16 h, (quantitative yield over two steps), (iii) HF.Pyr, THF/Pyr, rt, 15 min, 69%, (iv) DMTrCl, Pyr, 0°C, 16 h, 32%, (v) pyBOP, DIPEA, DMF, rt, 16 h, 37%, (vi) 2-Cyanoethyl *N*,*N*-diisopropylchlorophosphoramidite, DIPEA, DCM, rt, 16 h, 73%, (vii) Tetrazole, MeCN, rt, 20 h, (viii) I_2_, THF/Pyr/H_2_O, rt, 1 h, (ix) TCA, DCM, rt, 30 min (38% over three steps).

The sequences AA and GAA (Figure 1A) corresponding to the RlmJ RNA-substrate sequence were chosen to synthesize di and tri- nucleotides–SAM conjugates. We started our synthetic work by the synthesis of compound **7** (Scheme 2). First, the inosine was silylated in 2′-, 3′- and 5′-positions to afford compound **1**. A selective deprotection followed by the tritylation of the 5′-OH and activation of the O6-position using the BOP reagent (37) gave compound **4**. Then, the phosphitylation step produced compound **5** in 73% yield. Compound **5** in presence of the tetra-benzoylated adenosine **6** (36) provide the O^6^-(benzotriazolyl)-dinucleotide **7** in 38% yield over three steps (Scheme 2).

**Scheme 3.**
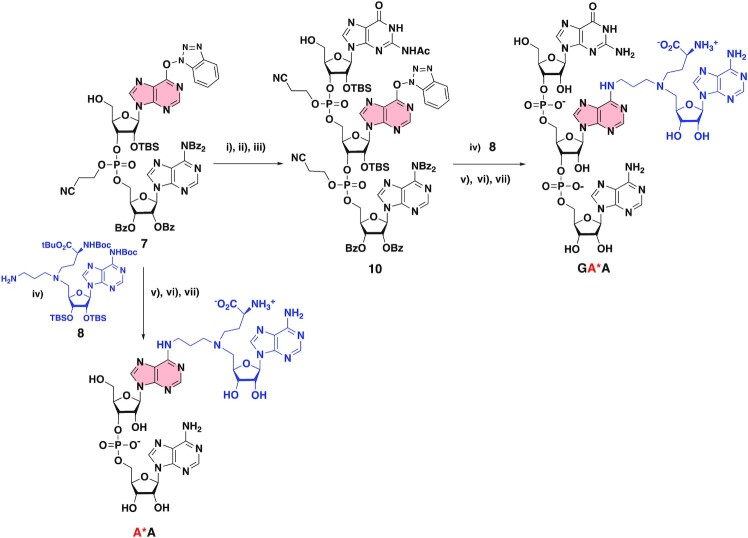
Synthesis of A*A and GA*A RNA–SAM conjugates. (i) Ac-G-CE-phosphoramidite, tetrazole, MeCN, rt, 20 h, (ii) I_2_, THF/Pyr/H_2_O, rt, 1 h, (iii) TCA, DCM, rt, 30 min, (49% over three steps); (iv) DIPEA 12 eq, DMF, rt, 24 h, (v) MeNH_2_, EtOH/H_2_O, rt, 24 h, (vi) CsF, MeOH, 60°C, 24 h; (vii) ZnBr_2_, MeNO_2_/iPrOH, rt, 24 h.

The key step of S_N_Ar reaction studied in this work was then optimized and efficient deprotection conditions were set up. The *O*^6^-(benzotriazolyl)-dinucleotide **7** was submitted to a S_N_Ar reaction with the compound **8** in the presence of diisopropylethylamine as a base (Scheme 3). During this step, the amine of the linker plays the role of the nucleophile in the S_N_Ar reaction but, also, in the deprotection step of the nucleic base. Addition of methylamine is necessary to complete the deprotection of the phosphotriester function and the nucleic bases. Cesium fluoride was used to remove the silyl ether groups and the removal of the Boc and the tBu protecting groups was performed using ZnBr_2_ salts in a mixture of isopropanol and nitromethane (37,38). The corresponding dinucleotide-SAM conjugate, A*A was obtained after reverse-phase HPLC purification and fully characterized by ^1^H,^31^P NMR (Supplementary Figures S8, S9) and mass spectrometry. A mass of 4.5 mg of A*A was obtained with an overall yield of 7% over four steps.

The synthesis of the trinucleotide GA*A started from dinucleotide **7** which was submitted to a phosphoramidite coupling in presence of the commercially available Ac-G-CE phosphoramidite leading to the formation of the *O*^6^-(benzotriazolyl)-trinucleotide **10** in 49% yield. The conjugate GA*A was then obtained after the addition of compound **8** followed by the three deprotection steps showing the possibility to functionalize an RNA at an internal position in the RNA sequence with the A^SAM^ linked to a nucleoside. The yields of the two last steps could not be determined since a pure GA*A compound has never been obtained. However, the synthesized quantity (0.7 mg) of GA*A and the purity were sufficient to obtain crystals and to solve the crystal structure of GA*A in complex with RlmJ.

**Scheme 4.**
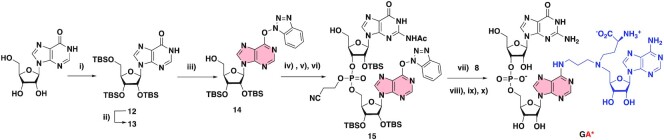
Synthesis of the GA* RNA-conjugate. (i) TBDMSCl, imidazole, DMF, 50°C, 18 h, 87%, (ii) PyBOP, DIPEA, DMF, rt, 16 h, 80%, (iii) TFA/H_2_O, THF, rt, 1h30, 63%, (iv) Ac-G-CE-phosphoramidite, tetrazole, MeCN, rt, 20 h, (v) I_2_, THF/Pyr/H_2_O, rt, 1 h, (vi) TCA, DCM, rt, 30 min (56% over three steps), (vii) DIPEA 12 eq, DMF, rt, 24 h, (viii) MeNH_2_, EtOH/H_2_O, rt, 24 h, (ix) CsF, MeOH, 60°C, 24 h (4% over three steps), (x) ZnBr_2_, MeNO_2_/iPrOH, rt, 24 h, 30%.

To introduce the SAM-cofactor at the 3′-end of the RNA, we first prepared the convertible nucleoside **14**. Inosine was silylated in 2′-, 3′- and 5′-positions to afford compound **12**, which was then activated using the PyBOP reagent to give compound **13**. The selective removal of the TBS group at the 5′-position was achieved in the presence of aqueous TFA to give compound **14** in an overall yield of 43% (Scheme 4). The *O*^6^-(benzotriazolyl)-dinucleotide **15** was then obtained in 56% yield using the strategy described for compound **7** and **10**. The S_N_Ar reaction with compound **8**, followed by three deprotection steps finally led to the formation of the dinucleotide GA*.

### Synthesis of a 13mer RNA–SAM conjugate by enzymatic ligation

**Scheme 5.**
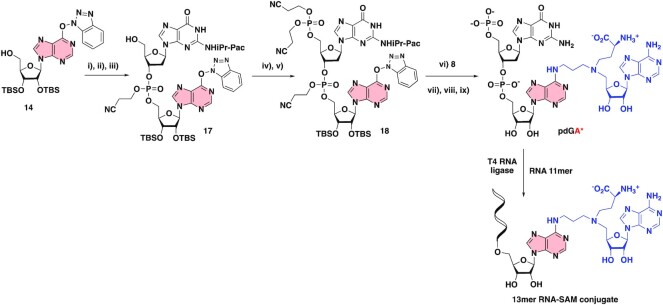
Synthesis of a 13mer RNA–SAM conjugate by enzymatic ligation. (i) iPr-Pac-dG-PCNE phosphoramidite, tetrazole, MeCN, rt, 20 h, (ii) I_2_, THF/Pyr/H_2_O, rt, 1 h, (iii) TCA, DCM, rt, 30 min (69% over three steps), (iv) (iPr)_2_NP(OCH_2_CH_2_CN)_2_, Tetrazole, MeCN, rt, 3 h, (v) I_2_, THF/Pyr/H_2_O, rt, 30 min; (85% over two steps), (vi) DIPEA, DMF, rt, 24 h, (vii) MeNH_2_, EtOH/H_2_O, rt, 24 h, (viii) CsF, MeOH, 60°C, 24 h (7% over three steps), (ix) ZnBr_2_, MeNO_2_/iPrOH, 4%. 11-mer RNA (GAACUCGCUGU, the sequence mimicking the substrate RNA of RlmJ, was synthesized by SPS).

The next objective was to synthesize RNA–SAM conjugates with an RNA of more than three nucleotides. An enzymatic ligation catalyzed by the T4 RNA ligase was used in the presence of a dinucleotide covalently linked to the A^SAM^ (pdGA*) and a 11 mer RNA (Scheme 5). This ligation was performed with a synthetic RNA molecule of 11 nucleotides corresponding to the 5′-part of the RNA substrate of RlmJ (Figure 1). The T4 RNA ligation requires a dinucleotide phosphorylated in 5′ position and an RNA (39). The dinucleotide **17** was obtained from compound **14** in three steps in an overall yield of 69% and then submitted to a phosphorylation step, affording the dinucleotide **18** in 85% yield over two steps (Scheme 5). Addition of the SAM analogue **8**, followed by the deprotection steps, gave the dinucleotide pdGA* conjugate in a quite low yield (7% yield for the two first deprotection steps and 4% yield for the last one). However, the quantity of the phosphorylated dinucleotide (0.4 mg) was sufficient to perform the T4 RNA ligation. The SAM-RNA conjugate was purified by size exclusion chromatography to remove excess of pdGA* and characterized by mass spectrometry.

### CA is able to bind RlmJ similarly to the SAM cofactor

**Figure 3.**
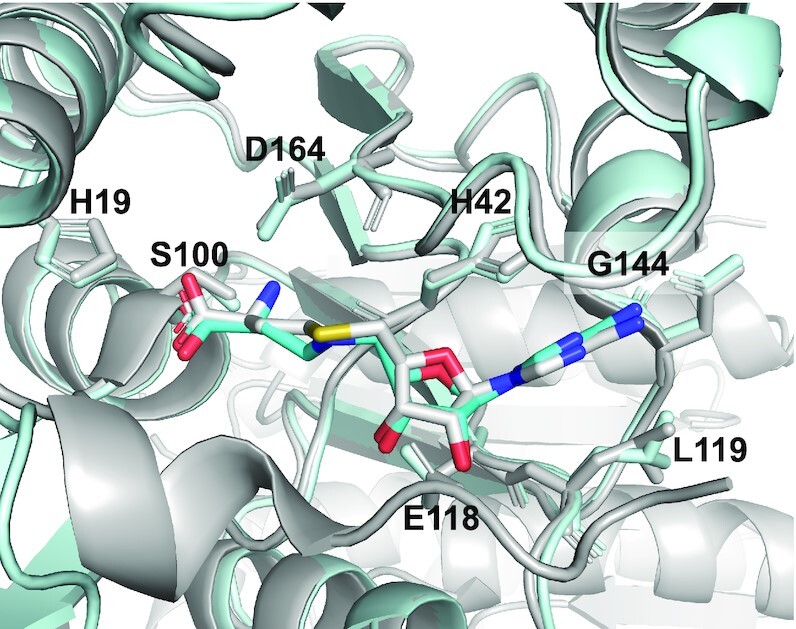
Crystal structure of RlmJ/CA complex. Superimposition of the crystal structure of RlmJ bound to **CA** (cyan) and to SAH (grey, PDB 6QE5). Side chains of conserved residues interacting with **CA** and SAH are drawn as sticks.

We first investigated whether **CA** could be accommodated in the RlmJ active site similarly to the SAM cofactor. The crystal structure of RlmJ bound to **CA** was refined to 2.1 Å (Table S1) and revealed a similar binding mode to that of the natural cofactor (Figure 3). **CA** and SAH exhibit the same conformations and positioning of both the methionine chain and the adenosine moiety in the active site of RlmJ. Both ligands bind in a pocket lined with RlmJ hydrophobic residues and form hydrogen bonds to conserved residues, namely H19, H42, S100 and D164 via the methionine part and E118 and G144 via the adenosine moiety of **CA**. The adenine ring is moreover sandwiched between H42 and L119. This structure therefore validates the use of this SAM analogue for the design of RNA–SAM conjugates presented in this paper.

### Use of RNA–SAM conjugates to solve crystal structures with RlmJ

Crystallization assays of RlmJ were performed in the presence of A*A, GA*, GA*A or the 13mer RNA–SAM conjugates in various RlmJ/compound ratios. Crystals were obtained for GA* and GA*A bound to RlmJ at a RlmJ/ GA* ratio of 1/2 and RlmJ/GA*A ratio of 1/8. The GA*A conjugate density was not well-defined with RlmJ/GA*A ratios <1/8. The structures of RlmJ bound with GA* or GA*A were refined to 2.3 Å and 1.6 Å, respectively (Supplementary Table S1). Both structures displayed well-defined ligand density in the active site, which facilitated fitting of both conjugates (Supplementary Figure S37). RlmJ displays a class I MTase Rossmann-fold with a helical subdomain inserted at residues 47–98, similar to what was previously described (15,24).

**Figure 4.**
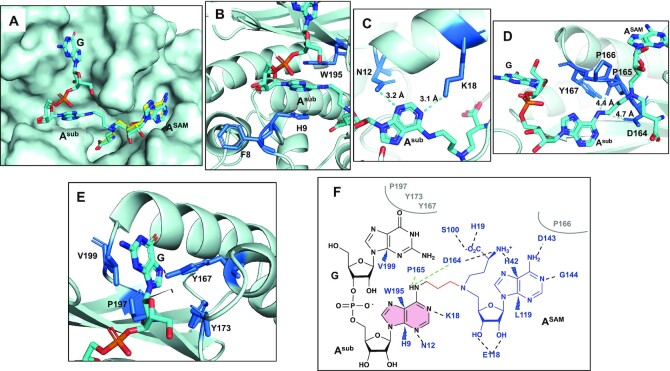
Crystal structure of RlmJ/GA* complex. (**A**) GA* on the surface of RlmJ, **CA** in the RlmJ/**CA** complex is represented as yellow sticks. (**B**) Stacking of the A^sub^ between the strictly conserved aromatic residues W195 and H9 of RlmJ. (**C**) A^sub^ is involved in hydrogen bonds with the strictly conserved residues N12 and K18 of RlmJ. (**D**) Interactions between the catalytic _164_DPPY_167_ motif of RlmJ and A^sub^. (**E**) Interactions of the guanosine (G) of GA* with RlmJ. (**F**) Outline of RlmJ/GA* interactions from a LigPlot analysis (40), dashed lines in black indicate residues in RlmJ that form hydrogen bonds with GA* in the crystal structure, dashed lines in green indicate residues in RlmJ that could form hydrogen bonds with the ligand if the N6-nitrogen is not alkylated (which is the case in the natural substrate), small dark blue lightnings highlight residues in RlmJ involved in strong hydrophobic contacts with GA* and residues involved in formation of the binding pocket environment are shown in grey.

In the RlmJ/GA* structure, the A^SAM^ moiety is superimposable with the RlmJ-bound conformation of SAH, A^sub^ (corresponding to A2030 in the *E. coli* 23S rRNA) is bound in the presumed substrate binding site, and the neighbouring guanosine (corresponding to G2029 in the *E. coli* 23S rRNA) binds in a pocket on the surface of RlmJ next to the catalytic site (Figure 4A). The N6-atom of A^sub^ is positioned, through the alkyl chain of the linker, at 3.0 Å away from the carbon corresponding to the Cϵ-atom of the methionine moiety in the SAM cofactor. Such a distance, in two non-linked moieties, would allow for an S_N_2 methyl transfer to the N6-atom. A^sub^ of GA* is involved in an intricate network of interactions with strictly conserved residues of RlmJ (Supplementary Figure S38) that reveals the RlmJ active site. A^sub^ is indeed π-stacked between the aromatic residues, H9 and W195 (Figure 4B), and is stabilised by hydrogen bonds from its N1- and N3-atoms to the side chains of the K18 and N12 residues, respectively (Figure 4C). The classical m^6^A MTase catalytic ^164^DPPY^167^ motif (D/N-PP-Y/F/W) also participates to the binding of GA* (Figure 4D). D164 forms a hydrogen bond of 2.7 Å to A^SAM^ and we propose that a hydrogen bond between D164 and the N6-atom of A2030 would form in the case of the non-alkylated RNA substrate (Figure 4F). This hydrogen bond cannot be formed in this structure due to the N6-alkylation of A^sub^ in GA*. Mutations of K18 and D164 to alanine were previously shown to abolish RlmJ activity, confirming their involvement in RNA binding and catalysis. P165 in the ^164^DPPY^167^ motif, through its carbonyl group, is also likely to form a second hydrogen bond to the N6-atom of A^sub^ (Figure 4D). In addition, P166 makes hydrophobic contacts with A^SAM^ (Figure 4D), and Y167 is engaged in hydrophobic interactions with the guanine ring of GA* (Figure 4E). Y167 is not stacked with A^sub^ in contrast to what was previously observed for F187 of the catalytic NPPF motif of METTL16 (13). The same ‘non-stacking’ position of Y167 is found in all RlmJ structures and appears important for stabilisation of the catalytic loop conformation, rather than for the substrate adenine position. Y167 also—along with residues V199, Y173 and P197—provides a hydrophobic surface near the catalytic pocket where the guanosine is positioned (Figure 4E). Taken together, each nucleic base of the GA* conjugate is involved in hydrophobic interactions with RlmJ residues, and both adenines, A^SAM^ and A^sub^, form hydrogen bonds to conserved residues in RlmJ (Figure 4F).

**Figure 5.**
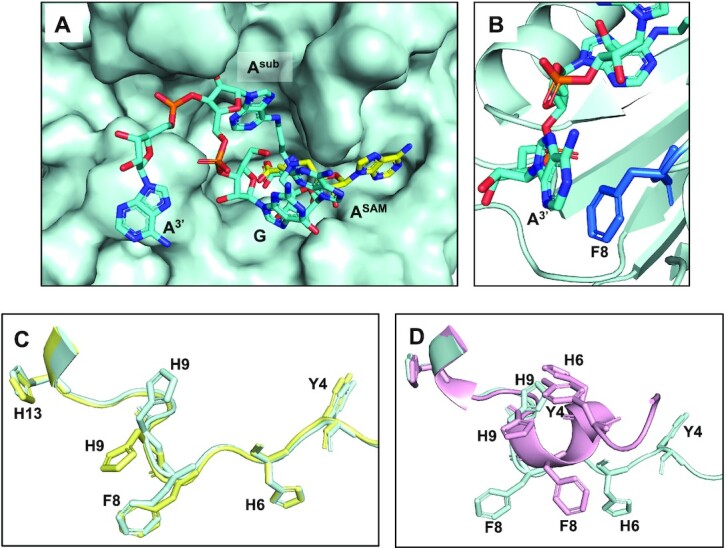
Crystal structure of the RlmJ/GA*A complex and analysis of the conformations of the N-terminal tail of RlmJ. (**A**) GA*A on the surface of RlmJ, the **CA** in the RlmJ/**CA** complex is represented as yellow sticks. (**B**) Stacking of the adenine at the 3′-end (A^3^′) of GA*A to F8 that is conserved as an aromatic residue (F or Y). (**C**) Superimposition of the N-terminal tail of RlmJ (residues 1–14) in the crystal structure of RlmJ in complex with **CA** (in yellow) and with GA* (in cyan). Conserved aromatic residues are drawn as sticks. (**D**) Superimposition of the N-terminal tail of RlmJ (residues 1–14) in the crystal structure of RlmJ in complex with GA* (in cyan) and with SAH (in pink, PDB 6QE5). Conserved aromatic residues are drawn as sticks.

In the RlmJ/GA*A structure, the ligand is folded via π-stacking of the A^SAM^ moiety with the guanine moiety of GA*A (Figure 5A). Compared to SAH, only the methionine part of **CA** is well-positioned in the active site of RlmJ. A^sub^ is pushed out of the active site and unable to form the stabilising interactions with H9, N12, K18, and W195, observed for the GA* conjugate. The GA*A conjugate is maintained bound to RlmJ through correct binding of the methionine part and the stacking of the adenosine at the 3′-end (A^3^′ in Figure 5, corresponding to A2031 in the *E. coli* rRNA) with the conserved residue F8 (Figure 5B). Such a folded conformation, which is not biologically relevant, was also previously observed for some bisubstrate adenosine-SAM analogues, caused by π-stacking between the two adenine rings during the process of crystallization (15). A folded conformation was not found for the GA* conjugate, suggesting that the formation of such a folded conformation depends on the RNA length and sequence. Therefore, synthesizing conjugates with different RNA lengths and sequences seems to be required to increase the chance of crystallizing a complex between a m^6^A RNA MTase and an RNA–SAM conjugate able to provide information about the RNA recognition by the MTase.

The N-terminal tail of RlmJ is flexible and includes the strictly conserved aromatic residues Y4, H6, F8 and H9. F8 π-stacks with A^3^′ in the GA*A-bound RlmJ structure (Figure 5B) whereas H9 π-stacks with A^sub^ in the GA*-bound RlmJ structure (Figure 4B). Interestingly, H9 swaps conformation from solvent exposed in the RlmJ/**CA** structure, to buried in the RlmJ/GA* structure (Figure 5C), from where it can π-stack with A^sub^. The positions of Y4 and H6 across RlmJ structures presented in this paper (Figure 5C) suggest an involvement in RNA nucleotide stacking, probably in the loop of the RNA substrate. Indeed, mutation of Y4 for an alanine or H6 for an aspartate abolished MTase activity (24). The N-terminal tail of RlmJ displays vastly different conformations in various crystal structures. In the apo form (PDB 4BLU), in the structures with SAM-adenosine bisubstrates (PDB: 6QDX and 6QE0) and in the three structures presented here, the tail is located on the surface of the protein and is devoid of any secondary structure. In structures of RlmJ bound to SAH (PDB: 6QE5) or SAH and AMP (PDB: 4BLW), a short 3_10_ helix is formed in the tail that packs against SAM and SAH (Figure 5D). In this ‘closed’ conformation of the tail, H9 is unable to π-stack with the RNA-substrate adenine due to steric clashes with H6 (Figure 5D). This conformation, if it exists in solution, is unlikely to be active. The crystal structure of RlmJ/SAM (PDB 4BLV), contains two monomers of RlmJ in the asymmetric unit. One monomer of SAM-bound RlmJ displays the ‘closed’ conformation, while the second one exhibits the open conformer with residues 1–8 adopting an extended conformation with no helix present. This suggests that, in the SAM-bound state, RlmJ is capable of populating these different conformations, at least in the crystal, with the binding of the RNA substrate requiring or selecting the open conformation of the N-terminal tail to clamp the RNA in the RlmJ active site.

### Deciphering the RNA recognition mode and methyl transfer used by RlmJ

**Figure 6.**
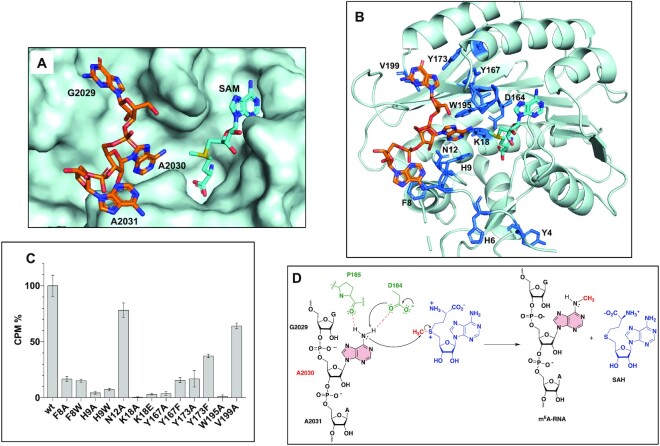
Model of a trinucleotide bound to RlmJ in position to be N6-methylated on the central adenosine. (**A**) RlmJ surface shown in pale cyan. (**B**) RlmJ secondary structure shown in pale cyan and side chains of residues interacting with the trinucleotide (in orange) shown as sticks. The model was built from the crystal structures of RlmJ/GA* and RlmJ/GA*A. (**C**) Methyltransferase assays of RlmJ variants mutated in the RNA binding interface. (**D**) Proposed mechanism of methyl transfer catalyzed by RlmJ.

From the structures of RlmJ bound to GA* and GA*A, we constructed a model of RlmJ bound to the SAM cofactor and a trinucleotide RNA covering ^2029^G**A**A^2031^ of the *E. coli* 23S rRNA sequence (Figure 6A, B). This model kept the structures of RlmJ and the GA dinucleotides of the RlmJ/GA* complex and that of A^SAM^ and the methionine chain of GA* to build the SAM. The third nucleotide, A2031, was added and positioned as the A^3^′ of GA*A in the RlmJ/GA*A structure. The phosphodiester backbone conformation of A2031 was adjusted to enable its linkage to A2030. To challenge the model, RlmJ residues in the GAA-binding interface (Figure 6A) were mutated and the mutant proteins were tested for MTase activity with the *E. coli* 23S rRNA hairpin (Figure 1A, Figure 6C). Mutated residues were selected according to the model as: (i) the two conserved aromatic residues in the N-terminal tail of RlmJ (F8, H9) that π-stack with A2031 and A2030, respectively, (ii) the three conserved residues (N12, K18 and W195) interacting with the target adenine A2030 and iii) the three residues (Y167, Y173 and V199) interacting with G2029. All mutant proteins are still folded and able to bind the SAM cofactor (Supplementary Figure S39). They all show a reduced MTase activity compared to the wild-type RlmJ, particularly for the mutations K18A and W195A that abolish the MTase activity. This confirms the contribution of F8 in the RNA-substrate binding, the involvement of H9, N12, K18 and W195 in the binding of the target adenine A2030 and the presence of a hydrophobic cavity made by the side chains of Y167, Y173 and V199 to bind G2029. Mutation of D164 to alanine was previously shown to abolish RlmJ MTase activity (24). Altogether, these data demonstrate that the SAM-RNA conjugates have been efficient to reliably investigate the RNA-binding determinants used by RlmJ to specifically recognize and modify its RNA substrate.

Based on this model, we propose here a mechanism for the methyl transfer reaction catalyzed by RlmJ (Figure 6D). The proper placement of adenosine 2030 is conditioned by π-π stacking with the aromatic side chains of H9 and W195 and by hydrogen bonds with N12 and K18. The N-terminal flexible tail is used to bind nucleosides in the loop of the 23S rRNA, F8 for A2031, H9 for A2030 and Y4 and H6 for two potential other nucleosides, explaining how the loop is bound by RlmJ. The N6 amino group of the A2030 is engaged in two hydrogen bonds, which enhance the nucleophilicity of the N6 and triggers the S_N_2 reaction into the electrophilic methyl group (Figure 6D).

## CONCLUSION

We have developed a method to synthesize RNA–SAM conjugates based on a convertible nucleoside strategy starting from O^6^-(benzotriazolyl)inosine. The O^6^-(benzotriazolyl)inosine can be introduced by phosphoramidite chemistry either at the 5′-end, 3′-end or an internal position of an oligonucleotide. The S_N_Ar reaction in the presence of a SAM analogue containing an alkyl linker was optimized and efficient deprotection conditions to provide fully deprotected dinucleotide- or trinucleotide-SAM conjugates were developed. We were also able to synthesize a 13mer RNA–SAM conjugate using an enzymatic ligation. The strategy, devised here, is chemo- and regio-selective and enables us to modify the size and the sequence of the RNA and to connect specifically the SAM cofactor to the adenine, which is methylated by m^6^A RNA MTases. Crystal structures of RlmJ/GA* and RlmJ/GA*A complexes have been solved and allowed us to construct a model of RlmJ bound to the SAM cofactor and a trinucleotide. This model is corroborated by site-directed mutagenesis and MTase activity assays and allows us to propose a mechanism for the methyl transfer reaction catalyzed by RlmJ. We have thus demonstrated here that RNA–SAM conjugates can be used to decipher RNA recognition by m^6^A RNA MTases. These structures were critical to obtain structural data impossible to obtain to date using fragments of the substrate RNA.

The conjugates described here can be used for any m^6^A MTases to probe their active site, by adapting the RNA sequence to the RNA substrate of the MTase of interest. In the future, chemistry that allows coupling of the cofactor to different positions in the RNA base or sugar, would facilitate the production of a full co-crystallization library of RNA-cofactor conjugates, matching each family of proteins catalyzing post-transcriptional modifications of RNA. Such a library would be a major advancement in the fields dealing with substrate recognition and enzymatic mechanisms of these protein families.

## DATA AVAILABILITY

Atomic coordinates and structure factors for the reported crystal structures have been deposited with the Protein Data bank under accession number 7P90, 7P9I, 7P8Q.

## Supplementary Material

gkac354_Supplemental_File
