# Supplementary material for: Synthesis of RNA-cofactor conjugates and structural exploration of RNA recognition by an m6A RNA methyltransferase
Source: Nucleic Acids Res. 2022 May 17;50(10):5793–806. doi: 10.1093/nar/gkac354 (PMC9178011; doi:10.1093/nar/gkac354)
Supplement: gkac354_Supplemental_File [file gkac354_supplemental_file.docx]

Supplementary data

|  |
| --- |

**Synthesis of RNA-cofactor conjugates and structural exploration of RNA recognition by an m^6^A RNA methyltransferase**

Vincent Meynier^1,†^, Laura Iannazzo^2,†^, Marjorie Catala^1^, Stephanie Oerum^1^, Emmanuelle Braud^2^, Colette Atdjian^2^, Pierre Barraud^1^, Matthieu Fonvielle^3^, Carine Tisné^$1^* and Mélanie Etheve-Quelquejeu^$2^*.

^1^ Expression Génétique Microbienne, UMR 8261, CNRS, Université Paris Cité, Institut de Biologie Physico-Chimique (IBPC), 75005 Paris, France

^2^ Laboratoire de Chimie et Biochimie Pharmacologiques et Toxicologiques, UMR 8601, CNRS, Université Paris Cité, 75006 Paris, France

^3^ Sorbonne Universités, UPMC, Sorbonne Paris Cité, Université Paris Cité, Centre de recherche des Cordeliers, 75006 Paris, France

[carine.tisne@cnrs.fr](mailto:carine.tisne@cnrs.fr)

[melanie.etheve-quelquejeu@u-paris.fr](mailto:melanie.etheve-quelquejeu@u-paris.fr)

**1. General information**

**Purification**

- Flash chromatography were done over silica gel (60 Å, 180-240 mesh; Merck, Darmstadt, Germany).
- Preparative HPLC were performed using a Reverse-phase HPLC system (Shimadzu, Marne-la-Vallée, France) with a reverse phase C-18 NUCLEOSIL column (250 mm × 21.2 mm, 5 µm) using a solvent system consisting of A: 50 mM aqueous NH_4_OAc pH 4.5 and B: MeCN (linear gradient from 0% B to 63% B in 30 min) at a flow rate of 15 mL/min and UV detection at 254 nm.

**Analysis**

- NMR spectra were recorded using Bruker spectrometers Bruker Advance II 500, and Bruker Advance III HD 400 (Bruker Biospin, Fällanden, Switzerland). Chemical shifts (δ) are reported in parts per million (ppm) and referenced to the residual proton or carbon resonance of the solvents: CDCl_3_ (δ 7.26), D_2_O (δ 4.79) or CD_3_OD (δ 3.31) for ^1^H and CDCl_3_ (δ 77.16) or CD_3_OD (δ 49.00) for ^13^C. Signals were assigned using 1D (^1^H and ^13^C) and 2D (HSQC, COSY and HMBC) experiments. NMR coupling constants (*J*) are reported in Hertz (Hz) and splitting patterns are indicated as follows: s (singlet), bs (broad singlet), d (doublet), t (triplet), q (quartet), dd (doublet of doublet), m (multiplet).
- High-resolution mass spectroscopy spectra (HRMS) were recorded with an ion trap mass analyser under electrospray ionization (ESI) in negative and positive ionization mode detection. HRMS were performed using Thermo Scientific LTQ Orbitrap XL (Thermo Scientific, Illkirch, France). Low-resolution mass spectroscopy spectra were obtained on a LCQ Advantage mass spectrometer (Thermo Scientific, Illkirch, France).

**2. Synthetic procedure and characterization**

Compound **6**^[[1]](#footnote-1)^ and **8**^[[2]](#footnote-2)^ were synthesized according to the procedure described in the literature.

**Compound 1**

Inosine (2.0 g, 7.45 mmol) was dissolved in DMF (20 mL) and di-*tert*-butylsilyl bis(trifluoromethanesulfonate) (2.67 mL, 8.19 mmol) was added dropwise at 0 °C. The reaction was stirred for 30 min at 0 °C and imidazole (2.53 g, 37.25 mmol) was added. The reaction was allowed to warm to room temperature and stirred for an additional hour. Then *tert*-butyl chloride (2.24 g, 14.9 mmol) and imidazole (1.01 g, 14.9 mmol) were added successively at 0 °C and the reaction was stirred at room temperature overnight. The reaction was diluted with EtOAc and washed five times with brine. The combined organic layers were dried over MgSO_4_, filtered and concentrated. The residue was purified by silica gel chromatography (eluent: cyclohexane/EtOAc 5:5) to provide the desired compound **1** as a white foam (3.9 g, quantitative yield). **^1^H NMR (500 MHz, CDCl_3_):** δ = 13.20 (bs, 1H, NH), 8.30 (s, 1 H, H8 or H2), 7.85 (s, 1 H, H2 or H8), 5.92 (s, 1 H, H1’), 4.53-4.52 (m, 1 H, H2’), 4.49-4.46 (m, 1 H, H5’), 4.35 (dd, *J* = 5.0, 9.7 Hz, 1 H, H3’), 4.23-4.18 (m, 1 H, H4’), 4.04-4.00 (m, 1 H, H5’), 1.05 (s, 9 H, *t*Bu), 1.02 (s, 9 H, *t*Bu), 0.91 (s, 9 H, *t*Bu), 0.14 (s, 3 H, Me^TBS^), 0.12 (s, 3 H, Me^TBS^). **^13^C NMR (126 MHz, CDCl_3_):** δ = 159.2 (Cq), 148.3 (Cq), 145.6 (C2 or C8), 138.2 (C2 or C8), 125.4 (Cq), 92.3 (C1’), 76.0 (C3’), 75.8 (C2’),74.8 (C4’), 67.8 (C5’), 27.5 (3 C, *^t^*Bu^TBS^), 27.1 (3 C, *^t^*Bu^TBS^), 25.9 (3 C, *^t^*Bu^TBS^), 22.8 (Cq*^t^*^Bu^), 20.4 (Cq*^t^*^Bu^), 18.3 (Cq*^t^*^Bu^), -4.2 (Me^TBS^), -4.8 (Me^TBS^). **HRMS (ESI) *m*/*z*:** [M + Na]^+^ Calcd for C_24_H_42_N_4_NaO_5_Si_2_ 545.2591; found: 545.2600.

**Compound 2**

To a solution of **1** (3.45 g, 6.6 mmol) in THF (60 mL), hydrogen fluoride pyridine complex (1.48 mL, 16.5 mmol) in pyridine (2.5 mL) was added at 0 °C and the mixture was stirred at room temperature for 15 min. The reaction was diluted with DCM and extracted with sat. NaHCO_3_. The organic layer was dried over anhydrous MgSO_4_, filtered and concentrated under reduced pressure. The crude residue was purified by column chromatography using DCM/MeOH (9/1) as the eluent to afford compound **2** as a white foam (1.74 g, 69%). **^1^H NMR (500 MHz, CDCl_3_):** δ = 8.39 (s, 1 H, H2 or H8), 8.12 (1 H, H2 or H8), 6.02 (d, *J* = 5.5 Hz, 1 H, H1’), 4.75-4.73 (m, 1 H, H2’), 4.28-4.27 (m, 1 H, H3’), 4.18-4.17(m, 1 H, H4’), 3.91-3.88 (m, 1 H, H5’), 3.79-3.76 (m, 1 H, H5’), 0.80 (s, 9 H, *t*Bu), -0.03 (s, 3 H, Me^TBS^), -0.14 (s, 3 H, Me^TBS^). **^13^C NMR (126 MHz, CDCl_3_):** δ = 158.8 (C=O), 149.6 (Cq), 147 (C2 or C8), 141.1 (C2 or C8), 126.1 (Cq), 90.6 (C1’), 87.0 (C4’), 77.8 (C2’), 72.4 (C3’), 63.0 (C5’), 26.1 (3 C, *t*Bu^TBS^), 18.9 (Cq*^t^*^Bu^), -4.8 (Me^TBS^), -5.2 (Me^TBS^). **HRMS (ESI) *m*/*z*:** [M + Na]^+^ Calcd for C_16_H_26_N_4_NaO_5_Si_2_ 405.1570; found: 405.1579.

**Compound 3**

Compound **2** (1.74 g, 4.5 mmol) was dissolved in pyridine (7 mL). 4,4’-Dimethoxytrityl chloride (1.69 g, 4.9 mmol) was added at 0 °C and the reaction was stirred at 0 °C for 16 h. The reaction was diluted in DCM, washed with brine, dried over anhydrous MgSO_4_, filtered and concentrated. The residue was purified by silica gel chromatography (eluent: DCM/MeOH 96:4) to provide the desired compound **3** as a white foam (1.01 g, 32 %). **^1^H NMR (500 MHz, CDCl_3_):** δ = 8.35 (s, 1 H, H8 or H2), 8.31 (s, 1 H, H8 or H2), 8.13-8.11 (m, 1 H, HAr), 7.54-7.51 (m, 1 H, HAr), 7.47-7.44 (m, 2 H, HAr), 7.43-7.41 (m, 2 H, HAr), 7.32-7.30 (m, 3 H, Ar-H), 7.27-7.24 (m, 3 H, HAr), 7.21-7.19 (m, 1 H, HAr), 6.81-6.79 (m, 4 H, HAr), 6.13 (d, *J* = 5.1 Hz, 1 H, H1’), 4.97-4.95 (m, 1 H, H2’), 4.38-4.36 (m, 1 H, H3’), 4.29-4.27 (m, 1 H, H4’), 3.76 (s, 6 H, 2 x O-CH_3_), 3.52 (dd, *J* = 3.0, 10.7 Hz, 1 H, H5’), 3.42 (dd, *J* = 3.7, 10.7 Hz, 1 H, H5’), 0.83 (s, 9 H, *t*Bu^TBS^), 0.00 (s, 3 H, Me^TBS^), -0.13 (s, 3 H, Me^TBS^). **^13^C NMR (126 MHz, CDCl_3_):** δ = 159.3 (Cq), 158.8 (2C, Cq), 151.7 (Cq), 145.2 (Cq), 144.6 (Cq), 143.9 (C2 or C8), 143.7 (C2 or C8), 135.6 (Cq), 135.5 (Cq), 130.2 (4 C, CAr), 129.1 (CAr), 128.2 (2 C, CAr), 128.1 (2 C, CAr), 127.2 (CAr), 125.1 (CAr), 125.0 (Cq), 120.8 (CAr), 120.1 (Cq), 113.4 (4 C, CAr), 108.8 (Ar-CH), 88.9 (C1’), 87.0 (Cq^DMT^), 84.6 (C4’), 76.2 (C2’), 71.7 (C3’), 63.4 (C5’), 55.4 (2 C, 2 Me^DMT^), 25.7 (3 C, *t*Bu^TBS^), 18.0 (Cq^TBS^), -4.8 (Me^TBS^), -5.0 (Me^TBS^). **HRMS (ESI) *m*/*z*:** [M + H]^+^ Calcd for C_37_H_45_N_4_O_7_Si 685.3058; found: 685.3044.

**Compound 4**

Benzotriazol-1-yloxytripyrrolidinophosphonium hexafluorophosphate (PyBOP) (689 mg, 1.55 mmol) and DIPEA (339 μL, 1.93 mmol) were added at 0 °C to a solution of compound **3** (888 mg, 1.29 mmol) in DMF (5 mL) and the reaction mixture was stirred at room temperature for 16 h. The residue was partitioned between ethyl acetate and brine. The organic layer was dried over anhydrous MgSO_4_, filtered and concentrated. The residue was purified by silica gel chromatography (cyclohexane/EtOAc 7:3) to provide the desired compound **4** (384 mg, 37%). **^1^H NMR (500 MHz, CDCl_3_):** δ = 8.35 (s, 1 H, H2 or H8), 8.31 (s, 1 H, H2 or H8), 8.13-8.11 (m, 1 H, HAr), 7.54-7.51 (m, 1 H, HAr), 7.47-7.44 (m, 2 H, HAr), 7.43-7.41 (m, 2 H, HAr), 7.32-7.30 (m, 3 H, HAr), 7.27-7.24 (m, 3 H, HAr), 7.21-7.19 (m, 1 H, HAr), 6.81-6.79 (m, 4 H, HAr), 6.13 (d, *J* = 5.1 Hz, 1 H, H1’), 4.97-4.95 (s, 1 H, H2’), 4.38-4.36 (m, 1 H, H3’), 4.29-4.27 (m, 1 H, H4’), 3.76 (s, 6 H, 2 Me^DMTr^), 3.52 (dd, *J* = 3.0, 10.7 Hz, 1 H, H5’), 3.42 (dd, *J* = 3.7, 10.7 Hz 1 H, H5’), 0.83 (2s, 9 H, *t*Bu^TBS^), 0.00 (s, 3 H, Me^TBS^), -0.13 (s, 3 H, Me^TBS^). **^13^C NMR (126 MHz, CDCl_3_):** δ = 159.3 (Cq), 158.8 (2 C, Cq), 151.7 (Cq), 145.2 (Cq), 144.6 (Cq), 143.9 (C2 or C8), 143.7 (C2 or C8), 135.6 (Cq), 135.5 (Cq), 130.2 (4 C, C^DMTr^), 129.1 (C^OBt^), 128.2 (2 C, C^DMTr^), 128.1 (2 C, C^DMTr^), 127.2 (C^DMTr^), 125.1 (C^OBt^), 125.0 (Cq), 120.8 (C^OBt^), 120.1 (Cq), 113.4 (4 C, C^DMT^), 108.8 (C^OBt^), 88.9 (C1’), 87.0 (Cq^DMTr^), 84.6 (C4’), 76.2 (C2’), 71.7 (C3’), 63.4 (C5’), 55.4 (2 C, 2 Me^DMTr^), 25.7 (3 C, *t*Bu^TBS^), 18.0 (C_q_^TBS^), -4.8 (Me^TBS^), -5.0 (Me^TBS^). **HRMS (ESI) *m*/*z*:** [M + H]^+^ Calcd for C_43_H_48_N_7_O_7_Si 802.3384; found: 802.3365.

**Compound 5**

The protected compound **4** (464 mg, 0.57 mmol) was dissolved in DCM (7 mL). The 2-Cyanoethyl N,N-diisopropylchlorophosphoramidite (516 µL, 2.28 mmol) and DIPEA (403 µL, 2.28 mmol) were added to the reaction mixture at 0 °C. The solution was stirred at room temperature for 16 h, diluted with DCM and washed with a saturated solution of NaHCO_3_ and brine. The organic layer was dried over anhydrous MgSO_4_, filtered and concentrated. The reaction was purified by silica gel chromatography neutralized with DIPEA (eluent: cyclohexane/EtOAc 7:3) to provide the two diastereoisomers **5** as a white foam (424 mg, 73 %). **^1^H NMR (500 MHz, CDCl_3_):** δ = 8.40-8.37 (2s, 1 H, H8 or H2), 8.29 (s, 1 H, H8 or H2), 8.14-8.12 (m, 1 H, HAr), 7.56-7.53 (m, 1 H, HAr), 7.46-7.43 (m, 4 H, HAr), 7.36-7.32 (m, 4 H, HAr), 7.26-7.24 (m, 2 H, HAr), 7.23-7.19 (m, 1 H, HAr), 6.82-6.79 (m, 4 H, HAr), 6.15-6.08 (2d, *J* = 6.1 Hz, 1 H, H1’), 5.02-5.01 (m, 1 H, H2’), 4.40-4.37 (m, 2 H, H3’ and H4’), 3.96-3.80 (2m, 2 H, CH_2_-O), 3.77 (s, 6 H, OCH_3_^DMTr^), 3.63-3.51 (m, 3 H, 2 CH and H5’), 3.38-3.35 (m, 1 H, H5’), 2.64-2.61 (m, 2 H, CH_2_-CN), 1.19-1.15 (m, 6 H, 2 Me), 1.05-1.04 (m, 6 H, 2 x Me), 0.76 (s, 9 H, *t*Bu^TBS^), -0.03 (s, 3 H, Me^TBS^), -0.19 (s, 3 H, Me^TBS^). **^13^C NMR (126 MHz, CDCl_3_):** δ = 159.1 (Cq), 158.7 (2 C, Cq), 154.1 (Cq), 151.6 (C2 or C8), 144.5 (Cq), 144.0 (C2 or C8), 143.6 (Cq), 135.6 (Cq), 135.5 (Cq), 130.3 (C^OBt^), 130.2 (4 C, C^DMTr^), 129.0 (Cq), 128.3-128.2 (2 C, C^DMTr^), 128.1-128.0 (2 C, C^DMTr^), 127.2 (C^DMTr^), 125.0 (C^OBt^), 120.7 (C^OBt^), 120.1 (Cq), 117.7 (Cq), 113.4-113.3 (4 C, C^DMTr^), 108.8 (C^OBt^), 88.9-88.7 (C1’), 87.0-86.8 (Cq^DMTr^), 84.5-84.1 (C4’), 75.7-75.6 (C2’), 72.9-72.8 (C3’), 63.3-63.1 (C5’), 58.9-58.8 (CH_2_-O), 55.4-55.3 (2 C, 2 Me^DMTr^), 43.5-43.0 (2 C, 2 CH), 25.7 (3 C, *t*Bu^TBS^), 24.9-24.7 (4 C, 4 Me), 20.6 (*C*H_2_-CN), 18.1 (Cq^TBS^), -4.5 (Me^TBS^), -5.0 (Me^TBS^). **^31^P NMR (202 MHz, CDCl_3_):** δ = 151.12, 149.27. **HRMS (ESI) *m*/*z*:** [M + H]^+^ Calcd for C_52_H_65_N_9_O_8_PSi 1002.4463; found: 1002.4445.

**Compound 7**

To a solution of phosphoramidite **5** (184 mg, 180 μmol) in MeCN (2 mL) was added tetrabenzoyl-adenosine **6** (70 mg, 100 μmol). The reaction mixture was stirred at room temperature for 30 min and a 0.45 M tetrazole solution in MeCN (2.2 mL, 1 mmol) was added. After stirring at room temperature for 20 h, a 0.1 M iodine solution in THF/H_2_O/Pyridine (75/2/20, 3.1 mL) was added. After 1 h, the reaction mixture was diluted with EtOAc, washed with water, a saturated solution of Na_2_S_2_O_3_ and brine. The organic layers were combined, dried over anhydrous MgSO_4,_ filtered and concentrated under vacuo. The residue was then stirred with a 0.18 M TCA solution in DCM (5.7 mL) at room temperature for 30 min. The reaction mixture was diluted with DCM and the organic layer washed with a saturated solution of NaHCO_3_ and brine, dried over anhydrous MgSO_4_, filtered and evaporated. The residue was purified by silica gel chromatography using DCM/MeOH (98/2) as eluent to afford compound **7** (50 mg, 38%). **^1^H NMR (500 MHz, CDCl_3_):** δ = 8.71-8.70 (m, 1 H), 8.50-8.46 (m, 1 H), 8.41-8.38 (m, 2 H), 8.15-8.13 (m, 1 H), 8.01-7.97 (m, 2 H), 7.92-7.89 (m, 2 H), 7.86-7.82 (m, 4 H), 7.56-7.33 (m, 15 H), 6.63-6.60 (m, 1 H), 6.40-6.28 (m, 1 H), 6.25-6.10 (m, 2 H), 5.23-5.17 (m, 1 H), 5.10-5.02 (m, 1 H), 4.77-4.73 (m, 1 H), 4.65-4.57 (m, 3 H), 4.37-4.21 (m, 2 H), 3.96-3.77 (m, 2 H), 2.79-2.75 (m, 2 H), 0.70 (2s, 9 H), -0.15 (2s, 3 H), -0.34 (s, 3 H). **^31^P NMR (202 MHz, CDCl_3_):** δ = -1.52, -2.59. **HRMS (ESI) m/z:** [M + H]^+^ Calcd for C_63_H_61_N_13_O_15_PSi 1298.3917; found: 1298.3894.

**Compound 9**

SAM analogue **8** (69 mg, 74 μmol) and DIPEA (132 μL, 756 μmol) were added at 0 °C to a solution of compound **7** (82 mg, 63 μmol) in DMF (2 mL) and the reaction mixture was stirred at room temperature for 24 h. After concentration, the residue was dissolved in a 5 M solution of MeNH_2_ (EtOH/H_2_O, 1/1) (2.5 mL) and the reaction was stirred at room temperature for 24 h and concentrated. The residue was then dissolved in MeOH (10 mL) and CsF (1.9 g, 12.6 mmol) was added. The reaction mixture was stirred at 60 °C for 24 h. After concentration under vacuo, the residue was purified by reverse-phase HPLC. The appropriate fractions were collected and lyophilized, to give the dinucleotide **9** (9 mg of a mixture of compound **9** and *N-methylbenzamide, 7% yield of* ***9*** *(6 mg) taking account of the 1/5 ratio determined by ^1^H NMR*), *Note: The compound is inseparable from the N-methylbenzamide residue released during the deprotection step.*

**^1^H NMR (500 MHz, MeOD): ^1^H NMR (500 MHz, MeOD):** δ = 8.44 (s, 1 H), 8.20-8.07 (m, 5 H), 6.05-6.03 (m, 1 H), 5.92-5.89 (m, 2 H), 1 H masked in the residual peak of water, 4.69-4.63 (m, 3 H), 4.43-4.39 (m, 1 H), 4.36-4.33 (m, 1 H), 4.30-4.27 (m, 1 H), 4.23-4.21 (m, 3 H), 4.16-4.13 (m, 1 H), 4.03-4.01 (m, 1 H), 3.76-3.75 (m, 2 H), 3.61-3.52 (m, 2 H), 3.09-3.03 (m, 2 H), 2.97-2.95 (m, 1 H), 2.66-2.54 (m, 2 H), 2.05-2.01 (m, 1 H), 1.89-1.85 (m, 2 H), 1.81-1.70 (m, 2 H), 1.36 (s, 9 H), 1.34 (s, 9 H). **^31^P NMR (202 MHz, MeOD):** δ = -1.79. **HRMS (ESI) m/z:** [M + H]^+^ Calcd for C_46_H_67_N_17_O_17_P 1160.4638; found: 1160.4643.

**Compound A*A**

The crude dinucleotide **9** (9 mg, 7.6 μmol) was dissolved in a 5 M solution of ZnBr_2_ in a 1/1 (v/v) mixture of iPrOH/MeNO_2_ (608 μL, 3 mmol) and the reaction mixture was stirred at room temperature for 24 h. Water was added and the mixture was lyophilized. The residue was purified by reverse-phase HPLC and the appropriate fractions were collected and lyophilized, to give the expected dinucleotide **A*A** (4.5 mg of pure compound, 90% yield calculated from the 6 mg of **9**).). **^1^H NMR (500 MHz, MeOD):** δ = 8.49 (s, 1 H), 8.21 (s, 1 H), 8.18 (s, 1 H), 8.12 (s, 1 H), 8.10 (s, 2 H), 6.08 (d, *J* = 5 Hz, 1 H), 5.97 (d, *J* = 5 Hz, 1 H), 5.87 (d, *J* = 5 Hz, 1 H), 3 H masked in the residual peak of water, 4.70-4.68 (m, 1 H), 4.47-4.44 (m, 1 H), 4.40-4.38 (m, 1 H), 4.27-4.25 (m, 2 H), 3.81-3.80 (m, 2 H), 3.74-3.71 (m, 2 H), 3.67-3.65 (m, 2 H), 3.46-3.44 (m, 2 H), 3.25-3.22 (m, 2 H), 3.17-3.16 (m, 1 H), 2.82-2.74 (m, 3 H), 2.10-2.08 (m, 2 H), 1.90-1.86 (m, 2 H). **^31^P NMR (202 MHz, MeOD):** δ = -1.34. **HRMS (ESI) m/z:** [M + H]^+^ Calcd for C_37_H_51_N_17_O_15_P 1004.3482; found: 1004.3502. **Retention time:** 15.63 min.

**Compound 10**

Compound **7** (183 mg, 140 μmol) and Ac-G-PCNE phosphoramidite (250 mg, 266 μmol) were stirred for 30 min at room temperature in MeCN (2 mL). A 0.45 M tetrazole solution in MeCN (3.1 mL, 1.4 mmol) was added and the reaction mixture was stirred at room temperature. After 20 h, a 0.1 M iodine solution in THF/H_2_O/Pyridine (75/2/20, 4.2 mL) was added and stirred for 1 h. The reaction mixture was then diluted with EtOAc, washed with water, a saturated solution of Na_2_S_2_O_3_ and brine. The organic layers were combined, dried over anhydrous MgSO_4,_ filtered and concentrated under vacuo. The residue was then stirred with a 0.18 M TCA solution in DCM (7.8 mL) at room temperature for 30 min. The reaction mixture was diluted with DCM and the organic layer washed with a saturated solution of NaHCO_3_ and brine, dried over anhydrous MgSO_4_, filtered and evaporated. The residue was purified by silica gel chromatography using DCM/MeOH (96/4) as eluent to afford compound **10** (129 mg, 49%). **^1^H NMR (500 MHz, CDCl_3_):** δ = 9.01 (s, 1 H), 8.71-8.68 (m, 1 H), 8.57-8.56 (m, 1 H), 8.49-8.42 (m, 3 H), 8.12-8.10 (m, 1 H), 8.01-7.86 (m, 6 H), 7.83-7.79 (m, 4 H), 7.58-7.53 (m, 2 H), 7.49-7.44 (m, 4 H), 7.39-7.33 (m, 8 H), 6.60-6.55 (m, 1 H), 6.35-6.30 (m, 1 H), 6.21-6.10 (m, 2 H), 5.82-5.75 (m, 1 H), 5.25-5.12 (m, 2 H), 5.04-4.97 (m, 2 H), 4.76-4.73 (m, 1 H), 4.67-4.56 (m, 4 H), 4.39-4.30 (m, 4 H), 3.91-3.78 (m, 3 H), 3.67-3.63 (m, 1 H), 2.79-2.76 (m, 4 H), 2.28 (s, 3 H), 0.77 (2s, 9 H), 0.69 (2s, 9 H), 0.01 (2s, 3 H), -0.13 (2s, 6 H), -0.27 (2s, 3 H). **^31^P NMR (202 MHz, CDCl_3_):** δ = -2.09, -2.25, -2.35, -2.59. **MS (ESI) m/z:** [M + Na]^+^ Calcd for C_84_H_91_N_19_O_23_P_2_Si_2_ 1874.5446; found: 1874.5421.

**Compound 11**

SAM analogue **8** (76 mg, 83 μmol) and DIPEA (145 μL, 828 μmol) were added at 0 °C to a solution of compound **10** (129 mg, 69 μmol) in DMF (2 mL) and the reaction mixture was stirred at room temperature for 24 h. After concentration, the residue was dissolved in a 5 M solution of MeNH_2_ (EtOH/H_2_O, 1/1) (2.8 mL) and the reaction was stirred at room temperature for 24 h and concentrated. The residue was then dissolved in MeOH (10 mL) and CsF (2.1 g, 13.8 mmol) was added. The reaction mixture was stirred at 60 °C for 24 h. After concentration under vacuo, the residue was purified by reverse-phase HPLC. The appropriate fractions were collected and lyophilized, to give the trinucleotide **11** (9 mg of a mixture of compound **11** and *N-methylbenzamide, 7% yield of* ***11*** *(8 mg) taking account of the 1/5 ratio determined by ^1^H NMR*). **^1^H NMR (500 MHz, MeOD):** δ = 8.51 (s, 1 H), 8.41 (s, 1 H), 8.17 (s, 1 H), 8.16 (s, 1 H), 8.15 (s, 1 H), 8.14 (s, 1 H), 7.95 (s, 1 H), 6.10-6.09 (m, 2 H), 5.94 (d, *J* = 5 Hz, 1 H), 5.82 (d, *J* = 5 Hz, 1 H), 3 H masked in the residual peak of water, 4.72-4.68 (m, 3 H), 4.52-4.48 (m, 2 H), 4.37-4.34 (m, 1 H), 4.30-4.18 (m, 7 H), 4.07-4.04 (m, 1 H), 3.80-3.78 (m, 2 H), 3.62-3.54 (m, 2 H), 3.10-3.03 (m, 2 H), 2.86-2.82 (m, 2 H), 2.77-2.73 (m, 2 H), 2.05-2.02 (m, 2 H), 1.91-1.88 (m, 2 H), 1.40 (s, 9 H), 1.39 (s, 9 H). **^31^P NMR (202 MHz, MeOD):** δ = -1.68, -1.74. **HRMS (ESI) m/z:** [M + H]^+^ Calcd for C_56_H_79_N_22_O_23_P_2_ 1505.5112; found: 1505.5077.

**Compound GA*A**

Dinucleotide **11** (9 mg, 6 μmol) was dissolved in a 5 M solution of ZnBr_2_ in a 1/1 (v/v) mixture of iPrOH/MeNO_2_ (439 μL, 2.4 mmol) and the reaction mixture was stirred at room temperature for 24 h. Water was added and the mixture was lyophilized. The residue purified by reverse-phase HPLC and the appropriate fractions were collected and lyophilized, to give the trinucleotide **GA*****A** (0,7 mg of a mixture of **GA*A**, *N-methylbenzamide and a third unidentified compound) Note: The compound is inseparable from the N-methylbenzamide residue released during the deprotection step and as shown by HPLC, a third unidentified compound is present in the mixture*. **^1^H NMR (500 MHz, D_2_O):** δ = 8.38 (s, 1 H), 8.31 (s, 1 H), 8.06 (s, 1 H), 8.05 (s, 1 H), 7.94 (s, 1 H), 7.86 (s, 1 H), 7.83 (s, 1 H), 6.12-6.11 (m, 1 H), 5.99-5.98 (m, 1 H), 5.94-5.93 (m, 1 H), 5.83-5.82 (m, 1 H), 5.71-5.68 (m, 1 H), 4 H masked in the residual peak of water, 4.57-4.52 (m, 2 H), 4.42-4.40 (m, 2 H), 4.33-4.24 (m, 3 H), 3.92-3.89 (m, 1 H), 3.86-3.82 (m, 2 H), 3.75-3.71 (m, 1 H), 3.66-3.60 (m, 2 H), 3.42-3.40 (m, 1 H), 3.24-3.18 (2 H), 2.90-2.86 (m, 2 H), 2.77-2.70 (m, 2 H), 2.43-2.36 (m, 2 H), 2.18-2.13 (m, 2 H), 1.85-1.79 (m, 2 H). **^31^P NMR (202 MHz, D_2_O):** δ = -0.56, -0.58. **HRMS (ESI) m/z:** [M + Na]^+^ Calcd for C_47_H_62_N_22_O_22_P_2_Na 1371.3781; found: 1371.3738.

**Compound 12**

To a solution of inosine (1.0 g, 3.7 mmol, 1 eq) and imidazole (2.03 g, 29.8 mmol, 8 eq) in DMF (11 mL) was added TBDMSCl (2.25 g, 14.9 mmol, 4 eq) at 0 °C. The reaction mixture was then stirred at 50 °C for 16 h. The residue was partitioned between ethyl acetate and brine. The organic phase was washed with brine, dried over anhydrous MgSO_4_, filtered and concentrated. The residue was purified by silica gel chromatography (cyclohexane/EtOAc 5:5) to provide the desired compound **12** as a white foam (1.96 g, 87%).

**^1^H NMR (500 MHz, CDCl_3_):** δ = 12.92 (bs, 1 H, NH), 8.23 (s, 1 H, H8), 8.10 (s, 1 H, H2), 6.02 (d, *J* = 4.9 Hz, 1 H, H1’), 4.53-4.51 (m, 1 H, H2’), 4.31-430 (m, 1 H, H3’), 4.14 (s, 1 H, H4’), 4.01-3.98 (m, 1 H, H5’), 3.81-3.79 (m, 1 H, H5’), 0.96 (s, 9 H, *t*Bu^TBS^), 0.93 (s, 9 H, *t*Bu^TBS^), 0.81 (s, 9 H, *t*Bu^TBS^), 0.15-0.14 (m, 6 H, Me^TBS^), 0.11-0.10 (m, 6 H, Me^TBS^), -0.02 (s, 3 H, Me^TBS^), -0.18 (s, 3 H, Me^TBS^). **^13^C NMR (126 MHz, CDCl_3_):** δ = 159.4 (Cq), 149.0 (Cq), 144.6 (C2), 139.2 (C8), 125.2 (Cq), 88.4 (C1’), 85.6 (C4’), 76.7 (C2’), 71.9 (C3’), 62.6 (C5’), 26.2 (3 C, *t*Bu^TBS^), 26.0 (3 C, *t*Bu^TBS^), 25.8 (3 C, *t*Bu^TBS^), 18.7 (Cq^TBS^), 18.2 (Cq^TBS^), 18.0 (Cq^TBS^), -4.2 (Me^TBS^), -4.5 (Me^TBS^), -4.5 (Me^TBS^), -4.8 (Me^TBS^), -5.2 (2 C, 2 Me^TBS^). **HRMS (ESI) m/z:** [M + H]^+^ Calcd for C_28_H_55_N_4_O_5_Si_3_ 611.3480; found: 611.3482.

**Compound 13**

BOP (618 mg, 1.40 mmol) and DIPEA (304 μL, 1.74 mmol) were added at 0 °C to a solution of inosine **12** (711 mg, 1.16 mmol) in DMF (5 mL) and the reaction mixture was stirred at room temperature for 16 h. The residue was partitioned between ethyl acetate and brine. The organic layer was dried over anhydrous MgSO_4_, filtered and concentrated. The residue was purified by silica gel chromatography (cyclohexane/EtOAc 9:1) to provide the desired compound **13** as a white foam (677 mg, 80%).^[[3]](#footnote-3)^ **^1^H NMR (500 MHz, CDCl_3_):** δ = 8.61 (s, 1 H, H8 or H2), 8.37 (s, 1 H, H2 or H8), 8.10 (d, *J* = 10 Hz, 1 H, HAr), 7.50-7.48 (m, 1 H, HAr), 7.46-7.40 (m, 2 H, HAr), 6.14 (s, 1 H, H1’), 4.59-4.57 (m, 1 H, H2’), 4.34-4.32 (m, 1 H, H3’), 4.17-4.16 (s, 1 H, H4’), 4.05-4.02 (m, 1 H, H5’), 3.82-3.79 (m, 1 H, H5’), 0.95 (s, 9 H, *t*Bu^TBS^), 0.91 (s, 9 H, *t*Bu^TBS^), 0.79 (s, 9 H, *t*Bu^TBS^), 0.14 (2s, 6 H, Me^TBS^), 0.08 (2s, 6 H, Me^TBS^), -0.02 (s, 3 H, Me^TBS^), -0.19 (s, 3 H, Me^TBS^).

**Compound 14**

A 1/1 (v/v) mixture of TFA/H_2_O (3.5 mL, 46 mmol) was added at 0 °C to a solution of compound **13** (677 mg, 0.92 mmol) in THF (20 mL). The reaction mixture was stirred for 1h30 at room temperature. EtOAc and a saturated solution of NaHCO_3_ were added and the organic phase was washed with brine. The organic layer was dried over anhydrous MgSO_4_, filtered and concentrated. The residue was purified by silica gel chromatography using cyclohexane/EtOAc (7/3) as eluent to afford compound **14** (361 mg, 63%) as a white foam. **^1^H NMR (500 MHz, CDCl_3_):** δ = 8.41 (s, 1 H, H8 or H2), 8.24 (s, 1 H, H2 or H8), 8.13-8.12 (m, 1 H, HAr), 7.54-7.51 (m, 1 H, HAr), 7.44-7.26 (m, 2 H, HAr), 5.93 (d, *J* = 7.6 Hz, 1 H, H1’), 5.49- 5.46 (m, 1 H, OH), 4.98-4.96 (m, 1 H, H2’), 4.34-4.33 (m, 1 H, H3’), 4.18 (s, 1 H, H4’), 3.94- 3.91 (m, 1 H, H5’), 3.73-3.69 (m, 1 H, H5’), 0.94 (s, 9 H, *t*Bu^TBS^), 0.76 (s, 9 H, *t*Bu^TBS^), 0.12 (s, 3 H, Me^TBS^), 0.11 (s, 3 H, Me^TBS^), -0.12 (s, 3 H, Me^TBS^), -0.59 (s, 3 H, Me^TBS^). **^13^C NMR (126 MHz, CDCl_3_):** δ = 159.7 (Cq), 153.1 (Cq), 151.2 (C2 or C8), 145.5 (C2 or C8), 143.5 (Cq), 128.9 (CAr), 128.8 (Cq), 125.0 (CAr), 121.4 (Cq), 120.7 (CAr), 108.6 (CAr), 91.3 (C1’), 89.4 (C4’), 74.4 (C2’), 73.7 (C3’), 62.8 (C5’), 25.9 (3 C, *t*Bu^TBS^), 25.7 (3 C, *t*Bu^TBS^), 18.1 (Cq^TBS^), 17.8 (Cq^TBS^), -4.4 (2 C, Me^TBS^), -4.5 (Me^TBS^), -5.6 (Me^TBS^). **HRMS (ESI) m/z:** [M + Na]^+^ Calcd for C_28_H_43_N_7_O_5_Si_2_Na 636,2762; found: 636.2736.

**Compound 15**

To a solution of Ac-G-PCNE phosphoramidite (250 mg, 262 μmol) in MeCN (300 μL) was added compound **14** (85 mg, 138 μmol) in MeCN (2 mL). The reaction mixture was stirred for 30 min at room temperature and a 0.45 M tetrazole solution in MeCN (3 mL, 1.38 mmol) was added. After stirring at room temperature for 20 h, a 0.1 M iodine solution in THF/H_2_O/Pyridine (75/2/20, 4.1 mL) was added. After 1 h, the reaction mixture was diluted with EtOAc, washed with water, a saturated solution of Na_2_S_2_O_3_ and brine. The organic layers were combined, dried over anhydrous MgSO_4,_ filtered and concentrated under vacuo. The residue was then stirred with a 0.18 M TCA solution in DCM (7.6 mL) at room temperature for 30 min. The reaction mixture was diluted with DCM and the organic layer washed with a saturated solution of NaHCO_3_ and brine, dried over anhydrous MgSO_4_, filtered and evaporated. The residue was purified by silica gel chromatography using DCM/MeOH (96/4) as eluent to afford compound **15** as a white foam (91 mg, 56%). **^1^H NMR (500 MHz, CDCl_3_):** δ = 8.48 (s, 1 H), 8.45 (s, 1 H), 8.12-8.10 (m, 1 H), 7.96 (s, 1 H), 7.53-7.52 (m, 1 H), 7.48-7.45 (m, 2 H), 6.07-6.03 (m, 1 H), 5.80-5.69 (m, 1 H), 5.20-5.12 (m, 1 H), 5.06-4.98 (m, 1 H), 4.89-4.87 (m, 1 H), 4.83-4.80 (m, 1 H), 4.53-4.48 (m, 1 H), 4.40-4.31 (m, 4 H), 3.98-3.91 (m, 1 H), 3.82-3.77 (m, 1 H), 3.70-3.64 (m, 1 H), 2.86-2.77 (m, 2 H), 2.31 (s, 3 H), 0.93 (s, 9 H), 0.85 (s, 9 H), 0.74 (s, 9 H), 0.13 (s, 3 H), 0.11 (s, 3 H), 0.05 (s, 3 H), -0.07 (s, 3 H), -0.11 (s, 3 H), -0.23 (s, 3 H). **^31^P NMR (202 MHz, CDCl_3_):** δ = - 1.77, - 1.87. **HRMS (ESI) m/z:** [M - H]^-^ Calcd for C_49_H_73_N_13_O_13_PSi_3_ 1166.4496; found: 1166.4493.

**Compound 16**

SAM analogue **8** (84 mg, 156 μmol) and DIPEA (162 μL, 936 μmol) were added at 0 °C to a solution of compound **15** (91 mg, 78 μmol) in DMF (2 mL) and the reaction mixture was stirred at room temperature for 24 h. After concentration, the residue was dissolved in a 5 M solution of MeNH_2_ (EtOH/H_2_O, 1/1) (3.1 mL) and the reaction was stirred at room temperature for 24 h and concentrated. The residue was then dissolved in MeOH (10 mL) and CsF (2.3 g, 15.6 mmol) was added. The reaction mixture was stirred at 60 °C for 24 h. After concentration under vacuo, the residue was purified by reverse-phase HPLC. The appropriate fractions were collected and lyophilized, to give the dinucleotide **16** (4 mg, 4% over 3 steps). **^1^H NMR (500 MHz, MeOD):** δ = 8.54 (s, 1 H), 8.41 (s, 1 H), 8.37 (s, 1 H), 8.18 (s, 1 H), 7.92 (s, 1 H), 6.07 (d, *J* = 5 Hz, 1 H), 6.03 (s, 1 H), 5.80 (d, *J* = 5 Hz, 1 H), 4.70-4.67 (m, 2 H), 4.57-4.54 (m, 1 H), 4.46-4.40 (m, 3 H), 4.29-4.25 (m, 3 H), 4.19-4.16 (m, 2 H), 4.06-4.03 (m, 2 H), 3.78 (bs, 2 H), 3.63-3.57 (m, 1 H), 3.19-3.12 (m, 2 H), 2.94-2.82 (m, 3 H), 2.74-2.67 (m, 2 H), 1.94-1.89 (m, 2 H), 1.84-1.74 (m, 1 H), 1.59 (s, 9 H), 1.39 (s, 18 H). **^31^P NMR (202 MHz, MeOD):** δ = 0.05. **MS (ESI) m/z:** [M - H]^-^ Calcd for C_51_H_73_N_17_O_20_P 1274.49; found: 1274.53.

**Compound GA***

Dinucleotide **16** (4 mg, 3.3 μmol) was dissolved in a 5 M solution of ZnBr_2_ in a 1/1 (v/v) mixture of iPrOH/MeNO_2_ (268 μL, 1.3 mmol) and the reaction mixture was stirred at room temperature for 24 h. Water was added and the mixture was lyophilized. The residue was purified by reverse-phase HPLC and the appropriate fractions were collected and lyophilized, to give the dinucleotide **GA*** (1 mg, 30%). **^1^H NMR (500 MHz, D_2_O):** δ = 8.29 (s, 1 H), 8.04 (s, 1 H), 7.97 (s, 1 H), 7.90 (s, 1 H), 7.83 (s, 1 H), 6.04 (d, *J* = 5 Hz, 1 H), 5.86 (d, *J* = 5 Hz, 1 H), 5.66 (d, *J* = 5 Hz, 1 H), 2 H masked in the residual peak of water, 4.72-4.66 (m, 3 H), 4.63-4.60 (m, 1 H), 4.55-4.52 (m, 2 H), 4.42-4.37 (m, 3 H), 4.28 (bs, 1 H), 4.20-4.16 (m, 1 H), 3.97-3.93 (m, 2 H), 3.90-3.86 (m, 1 H), 3.82-3.79 (m, 1 H), 3.72-3.62 (m, 2 H), 3.57-3.52 (m, 3 H), 3.44-3.39 (m, 2 H), 2.48-2.42 (m, 1 H), 2.37-2.31 (m, 1 H). **^31^P NMR (202 MHz, D_2_O):** δ = -0.75. **MS (ESI) m/z:** [M - H]^-^ Calcd for C_37_H_49_N_17_O_16_P 1018.32; found: 1018.53. **Retention time:** 11.70 min

**Compound 17**

To a solution of iPr-Pac-G-PCNE phosphoramidite (500 mg, 0.53 μmol) in MeCN (600 μL) was added compound **14** (111 mg, 0.18 mmol) in MeCN (4 mL). The reaction mixture was stirred at room temperature for 30 min and a 0.45 M tetrazole solution in MeCN (4 mL, 1.8 mmol) was added. After stirring at room temperature for 20 h, a 0.1 M iodine solution in THF/H_2_O/Pyridine (75/2/20, 5.4 mL) was added. After 1 h, the reaction mixture was diluted with EtOAc, washed with water, a saturated solution of Na_2_S_2_O_3_ and brine. The organic layers were combined, dried over anhydrous MgSO_4,_ filtered and concentrated under vacuo. The residue was then stirred with a 0.18 M TCA solution in DCM (10 mL) at room temperature for 30 min. The reaction mixture was diluted with DCM and the organic layer washed with a saturated solution of NaHCO_3_ and brine, dried over anhydrous MgSO_4_, filtered and evaporated. The residue was purified by silica gel chromatography using DCM/MeOH (96/4) as eluent to afford compound **17** (147 mg, 69%). **^1^H NMR (500 MHz, CDCl_3_):** δ = 8.98 (s, 1 H), 8.54 (s, 1 H), 8.41 (s, 1 H), 8.08-8.05 (m, 2 H), 7.48-7.41 (m, 2 H), 7.13-7.09 (m, 2 H), 6.88-6.84 (m, 2 H), 6.26 (m, 1 H), 6.07 (bs, 1 H), 5.31-5.23 (m, 1 H), 4.84-4.80 (m, 1 H), 4.71-4.69 (m, 2 H), 4.55-4.26 (m, 7 H), 3.89-3.74 (m, 2 H), 3.64-3.61 (m, 1 H), 2.96-2.60 (m, 5 H), 1.17 (s, 6 H), 0.92 (s, 9 H), 0.84 (s, 9 H), 0.11 (s, 6 H), -0.04 (s, 3 H), -0.06 (s, 3 H). **^31^P NMR (202 MHz, CDCl_3_):** δ = -2.30, -2.61. **HRMS (ESI) m/z:** [M - H]^-^ Calcd for C_52_H_69_N_13_O_13_PSi_2_ 1170.4413; found: 1170.4413.

**Compound 18**

Compound **17** (147 mg, 0.12 mmol) was dissolved in MeCN (1 mL) and bis(2-cyanoethyl)diisopropylphosphoramidite (102 mg, 0.36 mmol) in MeCN (1 mL), followed by tetrazole (2.8 mL, 1.2 mmol, 0.45 M solution in MeCN) were added. The mixture was stirred at room temperature for 3 h and a 0.1 M solution of I_2_, in THF/H_2_O/Pyridine (75/2/20, 3.8 mL) was added. After being stirred at room temperature for 1 h, the mixture was diluted with EtOAc and washed successively with a saturated solution of Na_2_S_2_O_3_ and brine, dried over anhydrous MgSO_4,_ filtered and concentrated to dryness. The residue was purified by silica gel chromatography using DCM/MeOH (9/1) as eluent to afford compound **18** (134 mg, 85%). **^1^H NMR (500 MHz, CDCl_3_):** δ = 10.31 (d, *J* = 40 Hz, 1 H), 8.87 (s, 1 H), 8.52 (d, *J* = 15 Hz, 1 H), 8.41 (bs, 1 H), 8.07 (d, *J* = 10 Hz, 1 H), 7.96 (d, *J* = 20 Hz, 1 H), 7.52-7.47 (m, 1 H), 7.45-7.41 (m, 2 H), 7.13-7.10 (m, 2 H), 6.87 (d, *J* = 10 Hz, 2 H), 6.30-6.23 (m, 1 H), 6.09-6.06 (m, 1 H), 5.39-5.34 (m, 1 H), 4.82-4.76 (m, 1 H), 4.72-4.69 (m, 2 H), 4.56-4.49 (m, 1 H), 4.44-4.20 (m, 11 H), 3.63 (bs, 2 H), 3.17-3.08 (m, 1 H), 2.85-2.63 (m, 7 H), 1.17 (s, 6 H), 0.92 (s, 9 H), 0.85 (s, 9 H), 0.12 (2s, 3 H), 0.09 (2s, 3 H), 0.04 (2s, 3 H), -0.06 (2s, 3 H). **^31^P NMR (202 MHz, CDCl_3_):** δ = -2.06, -2.89. **HRMS (ESI) m/z:** [M + H]^+^ Calcd for C_58_H_78_N_15_O_16_P_2_Si_2_ 1358.4764; found: 1358.4740.

**Compound 19**

SAM analogue **8** (160 mg, 0.18 mmol) and DIPEA (355 μL, 2.04 mmol) were added at 0 °C to a solution of compound **18** (231 mg, 0.17 mmol) in DMF (3 mL) and the reaction mixture was stirred at room temperature for 24 h. After concentration, the residue was dissolved in a 5 M solution of MeNH_2_ (EtOH/H_2_O, 1/1) (6.8 mL) and the reaction was stirred at room temperature for 24 h and concentrated. The residue was then dissolved in MeOH (15 mL) and CsF (5.16 g, 34 mmol) was added. The reaction mixture was stirred at 60 °C for 24 h. After concentration under vacuo, the residue was purified by reverse-phase HPLC. The appropriate fractions were collected and lyophilized, to give the dinucleotide **19** (14 mg, 7% over 3 steps). **^1^H NMR (500 MHz, MeOD):** δ = 8.56 (s, 1 H), 8.13 (s, 2 H), 8.06 (s, 1 H), 7.95 (s, 1 H), 6.17 (t, *J* = 7.5 Hz, 1 H), 6.08 (d, *J* = 10 Hz, 1 H), 5.92-5.89 (m, 1 H), 2 H masked in the residual peak of water, 4.80-4.76 (m, 2 H), 4.71-4.69 (m, 1 H), 4.47-4.44 (m, 1 H), 4.40-4.37 (m, 1 H), 4.34-4.31 (m, 2 H), 4.25-4.24 (m, 1 H), 4.12-4.03 (m, 5 H), 3.70-3.66 (m, 1 H), 3.48-3.43 (m, 1 H), 3.39-3.36 (m, 2 H), 3.13-2.99 (m, 4 H), 2.48-2.41 (m, 2 H), 2.17-2.06 (m, 2 H), 1.39 (2s, 18 H). **^31^P NMR (202 MHz, MeOD):** δ = 1.15, -0.90. **HRMS (ESI) m/z:** [M + H]^+^ Calcd for C_46_H_68_N_17_O_20_P_2_ 1240.4301; found: 1240.4349.

**Compound pdGA***

Dinucleotide **19** (14 mg, 11 μmol) was dissolved in a 5 M solution of ZnBr_2_ in a 1/1 (v/v) mixture of iPrOH/MeNO_2_ (452 μL, 2.2 mmol) and the reaction mixture was stirred at room temperature for 24 h. Water was added and the mixture was lyophilized. The residue purified by reverse-phase HPLC and the appropriate fractions were collected and lyophilized, to give the dinucleotide **pdGA*** (0.4 mg, 4%). **^1^H NMR (500 MHz, D_2_O):** δ = 8.30 (s, 1 H), 8.04 (s, 1 H), 7.83 (s, 1 H), 7.80 (s, 1 H), 7.55 (s, 1H), 5.91 (d, *J* = 5 Hz, 1 H), 5.84-5.81 (m, 1 H), 5.70-5.69 (m, 1 H), 8 H masked in the residual peak of water, 4.56-4.54 (m, 1 H), 4.49-4.46 (m, 1 H), 4.35-4.32 (m, 1 H), 4.30-4.28 (m, 1 H), 4.23-4.20 (m, 1 H), 4.14-4.11 (m, 1 H), 4.00-3.98 (m, 1 H), 3.94-3.88 (m, 2 H), 3.60-3.57 (m, 1 H), 3.51-3.42 (m, 3 H), 3.25-3.19 (m, 1 H), 2.65-2.58 (m, 1 H), 2.39-2.30 (m, 2 H), 2.21-2.15 (m, 1 H), 1.98-1.94 (m, 1 H). **^31^P NMR (202 MHz, D_2_O):** δ = -1.22, -1.26. **MS (ESI) m/z:** [M + H]^+^ Calcd for C_37_H_52_N_17_O_18_P_2_ 1084.31; found: 1084.47. **Retention time:** 16.03 min.

**Synthesis of 11mer oligonucleotide**

**Solid-phase synthesis:** The 11mer RNA sequence (5’-GAACUCGCUGU-3’) was synthesized automatically (1 μmol scale) with the use of an H-2 GeneWorld DNA/RNA automated synthesizer (K&A, Laborgeraete GbR, Schaafheim, Germany). The commercially available phosphoramidites of A, C, U and G protected on the 5’- and 2’-hydroxy functions with DMTr and TBDMS groups, respectively, were used (Glen Research). The exocyclic amine functions were masked with acetyl protection for C and G nucleobases, benzoyl for A, and unprotected U (Glen Research). Universal support 500 (Glen Research) was used and all phosphoramidite monomers were dissolved in anhydrous acetonitrile to a concentration of 70 mM immediately prior to use. A 0.18 M trichloroacetic acid solution in DCM was used for the deprotection of DMTr group. The coupling in the presence of 5-ethylthio tetrazole activator (0.45 M in ACN, ChemGenes Coorporations) was performed three times, each step for 3 min. Capping was performed with the use of a mixture of Cap A (THF/Lutidine/Ac_2_O, 8/1/1, v/v/v, Merck) and Cap B (N-Methylimidazole in THF, 84/16; v/v). A 0.1 M iodine solution in THF/H_2_O/Pyridine (75/2/20, v/v/v) was used as the oxidizing agent. Stepwise coupling efficiencies were determined by automated dimethoxytrityl cation conductivity monitoring. After the last coupling, the DMTr group was removed and the support was washed and dried, and transferred to a screw cap glass vial.

**RNA deprotection (corresponding to 1.0 μmol):** The obtained CPG-bound oligomer was deprotected and cleaved from the support by treatment with a mixture of 28% aq. NH_3_ and 8 M ethanolic MeNH_2_ (2 mL, 1/1 v/v) for 1 h at 65 ºC. The supernatant was removed and the support was washed with ethanol (2 x 1 mL). The combined solutions were evaporated under vacuum. The 2'-*O*-TBS groups were removed with a solution of TEA·3HF/NMP/NEt_3_ (600 μL, 4/6/3 v/v/v) for 2.5 h at 65 ºC. The reaction was quenched by the addition of ethoxytrimethylsilane (300 μL) and the crude oligomer was precipitated using *tert*-butyl methyl ether (2 x 750 μL).

**Purification:** The fully deprotected oligoribonucleotide was purified by reverse-phase HPLC on C-18 column (4.6 x 250 mm, NUCLEOSIL, Macherey-Nagel) using a solvent system consisting of 50 mM aqueous NH_4_OAc (A) and CH_3_CN (B). The column was eluted with linear gradient from 0 to 63% of B in 45 min at a flow rate of 1.0 mL/min and UV detection at 254 nm. The appropriate fraction was collected and lyophilized to give 6.07 OD_260_ / 58 nmol of the desired oligomer. The quantity of oligoribonucleotide was determined by measuring the absorbance at 260 nm, assuming an extinction coefficient of 104 600 M^-1^ cm^-1^ (according to the <https://www.aatbio.com/tools/calculate-RNA-concentration>). The isolated RNA was analyzed by MS Calculated for C_104_H_130_N_40_O_76_P_10_: 3,464.5 found: 3,464.7 Da.

**Ligation of pdGA* with an 11 mer oligonucleotide and purification of the 13 mer RNA-SAM conjugate.**

The ligation reaction was performed at 30° C for 12 hours in 500 µl of Hepes buffer pH 7.5 (50 mM) containing the 11mer RNA (20 nmol), the **pdGA*** (200 nmol), T4 RNA ligase (0.3 mg), DMSO (10%), ATP (1 mM), MgCl2 (15 mM).

The 13mer RNA-SAM conjugate was purified by semi-preparative anion exchange chromatography (DNAPac PA-100; ThermoFisher Scientific) to remove excess of pdGA*. Final product was characterized by analytical DNAPac PA-100 anion exchange chromatography and LC-MS with 200 and 500 pmol of SAM-RNA conjugate, respectively.

Analytical DNAPAc analyse of 13mer SAM-RNA conjugate was performed by anion exchange chromatography (DNAPAc PA100; Dionex) with a linear gradient (25 mM to 2.5 M) of ammonium acetate (pH 8.0) containing 0.5% acetonitrile (v/v) applied between 5 and 20 min at a flow rate of 2 mL.min^-1^.

Mass spectrometry analysis of 13mer SAM-RNA conjugate was performed by LC-MS. Liquid chromatography (LC) was performed by anionic reverse-phase HPLC using a DNAPac RP column (4 µm; 2.1 x 50 mm; Thermo Fisher Scientific) at a flow rate of 300 µl.min-1 with a 0 to 50% MeOH linear gradient applied from 2 to 10 min in solution A [Solution A, triethylamine (15 mM) and 1,1,1,3,3,3-hexafluoro-2-propanol (400 mM)]. For mass spectrometry (MS), liquid chromatography was coupled to a LCQ Deca XP-Max mass spectrometer operating in the negative mode. Calculated for C_141_H_179_N_57_O_93_P_12_: 4,529.8 found: 4,529.2 Da.

Product was dissolved in RNAse free water (Sigma-Aldrich) and quantified by UV absorption at 260 nm (Ɛ^260nm^ = 122,600 M^-1^.cm^-1^).

**3. NMR spectra**

**Supplementary Figure S1**. 1H NMR spectrum of compound **5**.

**Supplementary Figure S2**. 13C NMR spectrum of compound **5**.


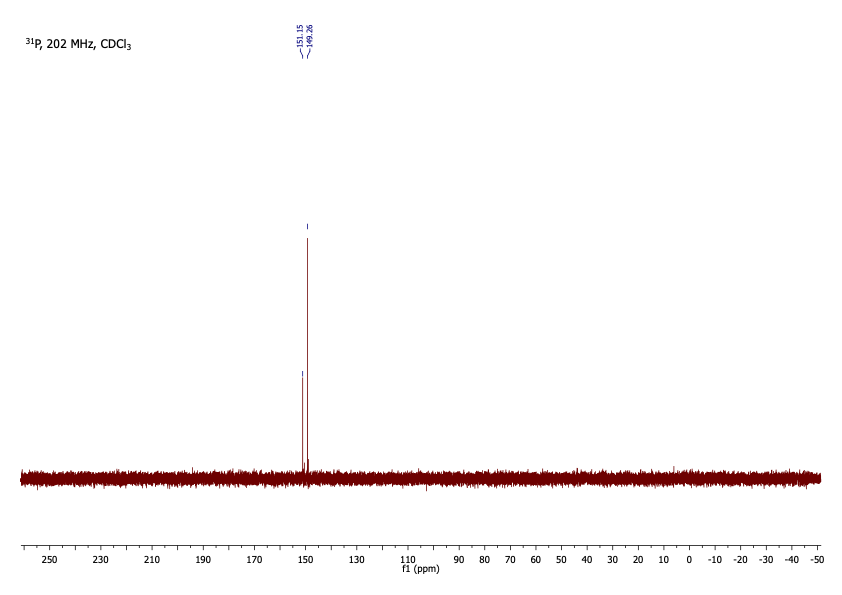


**Supplementary Figure S3**. 31P NMR spectrum of compound **5**

**Supplementary Figure S4**. 1H NMR spectrum of compound **7**.

**Supplementary Figure S5**. 31P NMR spectrum of compound **7**.


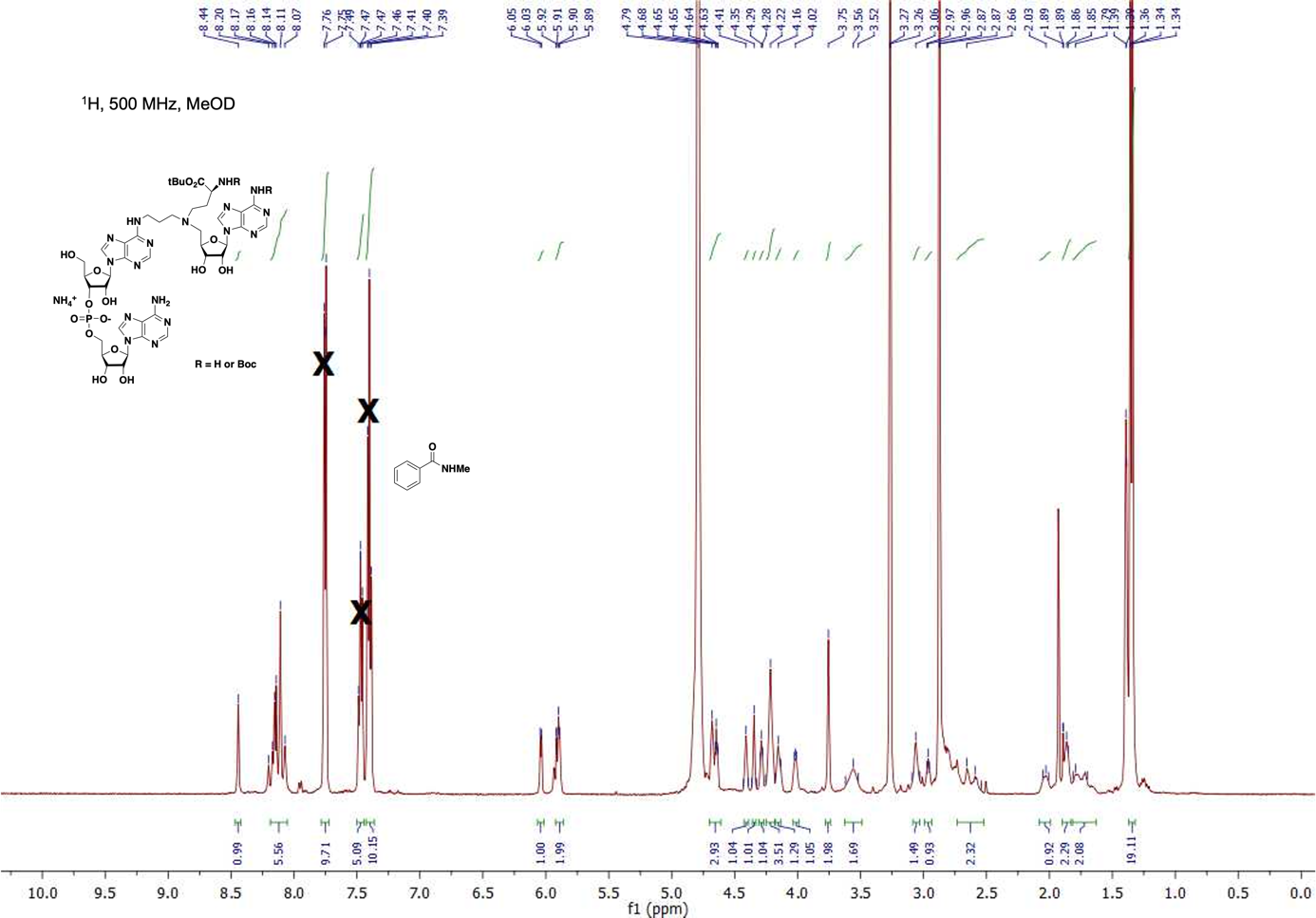


**Supplementary Figure S6**. 1H NMR spectrum of compound **9**

**Supplementary Figure S7**. 31P NMR spectrum of compound **9**

**Supplementary Figure S8**. 1H NMR spectrum of compound **A*A**

**Supplementary Figure S9**. 31P NMR spectrum of compound **A*A**

**Supplementary Figure S10**. 1H NMR spectrum of compound **10.**

**Supplementary Figure S11**. 31P NMR spectrum of compound **10.**

**Supplementary Figure S12**. 1H NMR spectrum of compound **11.**

**Supplementary Figure S13**. 31P NMR spectrum of compound **11.**


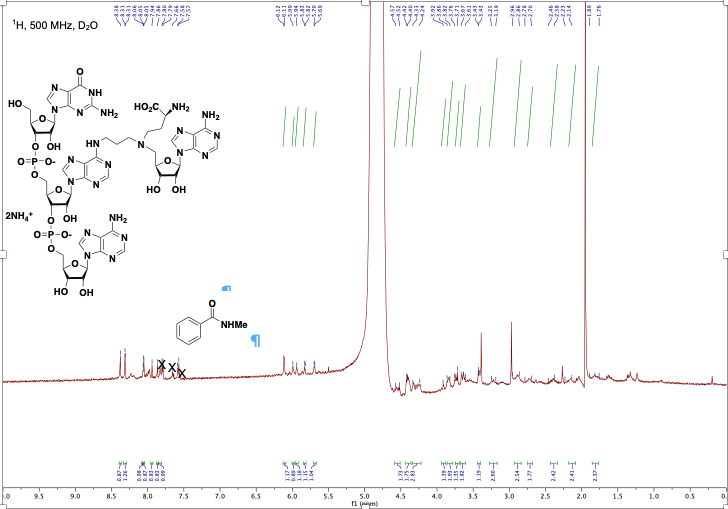


**Supplementary Figure S14**. 1H NMR spectrum of compound **GA*A.**

**Supplementary Figure S15**. 31P NMR spectrum of compound **GA*A.**

**Supplementary Figure S16**. 1H NMR spectrum of compound **15.**

**Supplementary Figure S17**. 31P NMR spectrum of compound **15.**

**Supplementary Figure S18**. 1H NMR spectrum of compound **16.**

**Supplementary Figure S19**. 31P NMR spectrum of compound **16.**

**Supplementary Figure S20**. 1H NMR spectrum of compound **GA*.**

**Supplementary Figure S21**. 31P NMR spectrum of compound **GA*.**

**Supplementary Figure S22**. 1H NMR spectrum of compound **17.**

**Supplementary Figure S23**. 31P NMR spectrum of compound **17.**


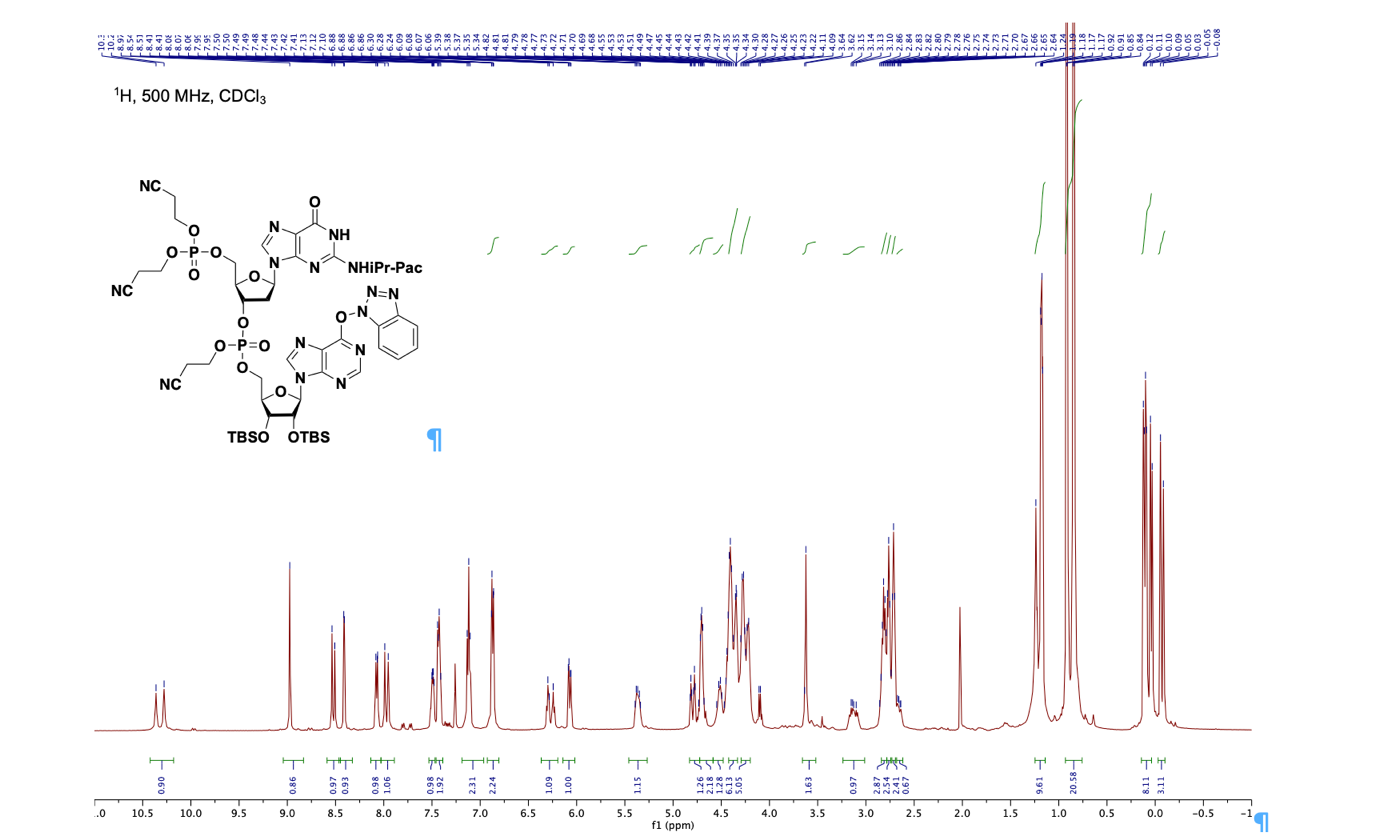


**Supplementary Figure S24**. 1H NMR spectrum of compound **18.**

**Supplementary Figure S25**. 31P NMR spectrum of compound **18.**

**Supplementary Figure S26**. 1H NMR spectrum of compound **19.**

**Supplementary Figure S27**. 31P NMR spectrum of compound **19.**

**Supplementary Figure S28**. 1H NMR spectrum of compound **pdGA*.**

**Supplementary Figure S29**. 31P NMR spectrum of compound **pdGA*.**

**4. Reverse-phase HPLC chromatogram of purified compounds**

C18 100-5 NUCLEOSIL (Macherey Nagel), 250 mm x 4.6 mm, 5 μm.

Flow: 1 mL/min; gradient: 0 to 63 % B in 30 min (A: NH_4_OAc; B: MeCN) at rt. Detection at 254 nm.

**Supplementary Figure S30**. Reverse-phase HPLC chromatogram of compound **GA*.**

C18 100-5 NUCLEOSIL (Macherey Nagel), 250 mm x 4.6 mm, 5 μm.

Flow: 1 mL/min; gradient: 0 to 63 % B in 30 min (A: NH_4_OAc; B: MeCN) at rt. Detection at 254 nm.

**Supplementary Figure S31**. Reverse-phase HPLC chromatogram of compound **A*A**

C18 100-5 NUCLEOSIL (Macherey Nagel), 250 mm x 4.6 mm, 5 μm.

Flow: 1 mL/min; gradient: 0 to 63 % B in 30 min (A: NH_4_OAc; B: MeCN) at rt. Detection at 254 nm.

**Supplementary Figure S32**. Reverse-phase HPLC chromatogram of compound **GA*A**

C18 100-5 NUCLEOSIL (Macherey Nagel), 250 mm x 4.6 mm, 5 μm.

Flow: 1 mL/min; gradient: 0 to 63 % B in 30 min (A: NH_4_OAc; B: MeCN) at rt. Detection at 254 nm.

**Supplementary Figure S33**. Reverse-phase HPLC chromatogram of compound **pdGA***

**5’-GAACUCGCUGU-3’: 11mer RNA**


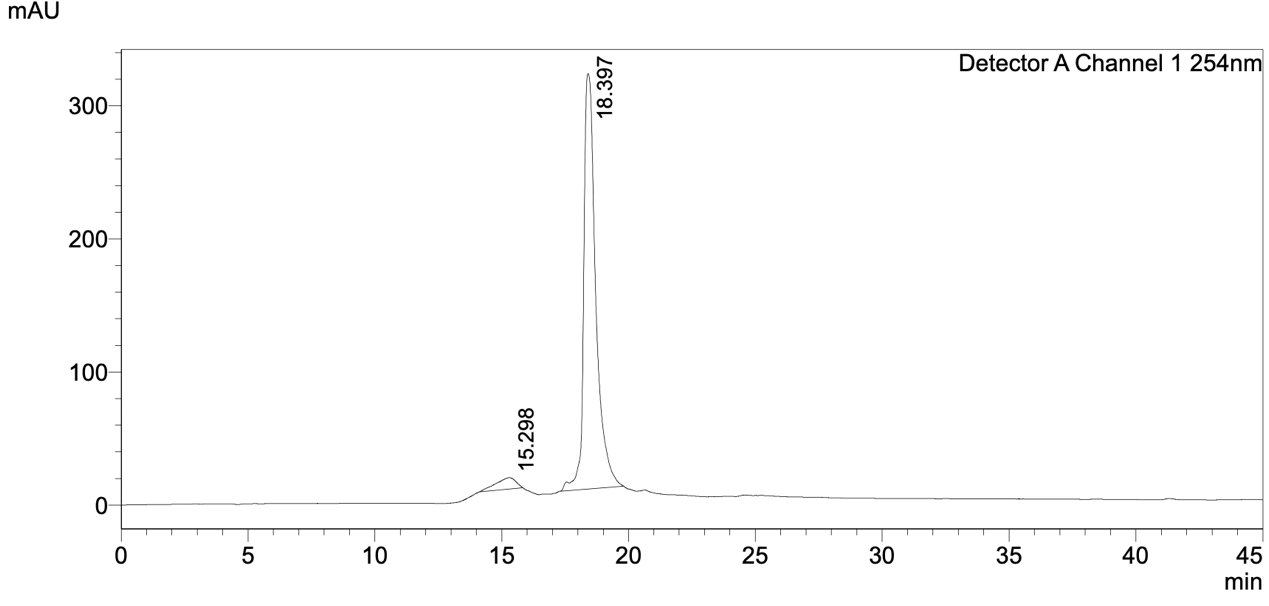


C18 100-5 NUCLEOSIL (Macherey Nagel), 250 mm x 4.6 mm, 5 μm.

Flow: 1 mL/min; gradient: 0 to 63 % B in 30 min (A: NH_4_OAc; B: MeCN) at rt.

**Supplementary Figure S34**. Reverse-phase HPLC chromatogram of the 11-mer RNA.


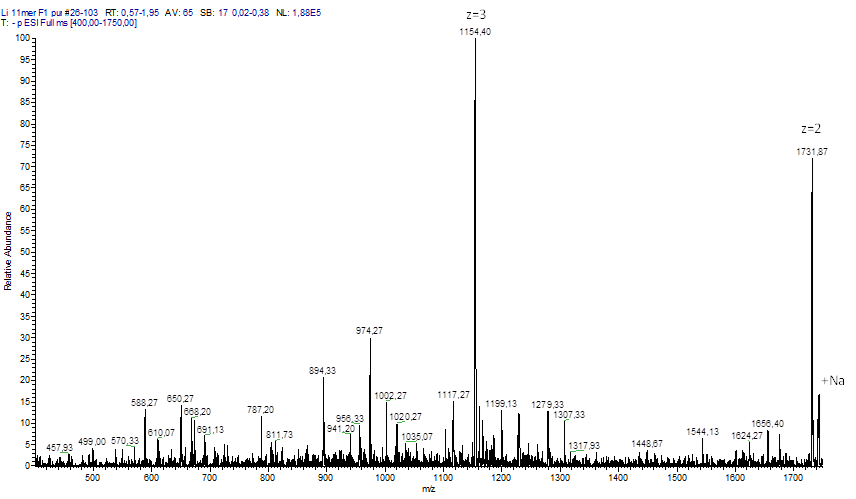


**Supplementary Figure S35**. Mass spectrum of the 11-mer RNA.


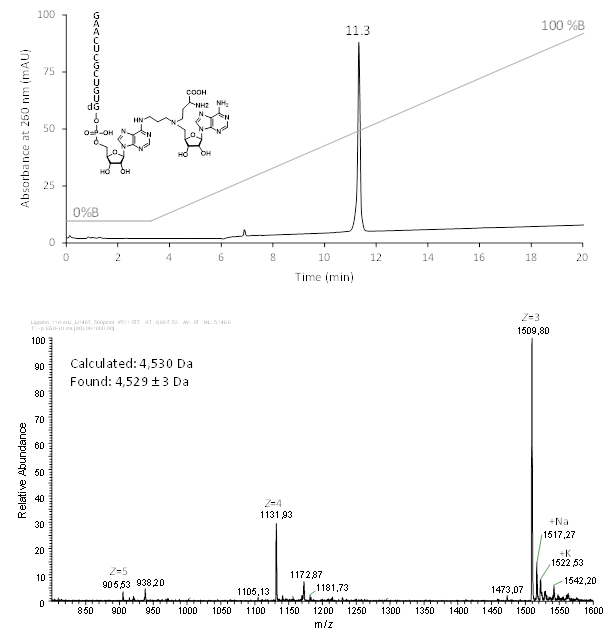


**Supplementary Figure S36**. Mass spectrum of the 13-mer RNA-SAM conjugate and reverse-phase HPLC chromatogram.

**Supplementary Figure S37**. Active site of the crystal structure of RlmJ in complex with A) CA, B) GA* and C) GA*A. The electron density map (2Fo-Fc) is shown for each RNA-SAM conjugate contoured at 1.0 σ in A and B and at 1.5 σ in C.

**Figure S38: Conserved residues in RlmJ.** Sequence alignment of a representative set of RlmJ sequences from proteobacteria with colour coding from Consurf.^[[4]](#footnote-4)^ The secondary structure of the *E. coli* RlmJ is indicated above the alignment. NCBI accession numbers of the sequences are as follows: *Escherichia coli* (NP_417956.1), *Salmonella enterica* (YP_001590613.1), *Haemophilus influenzae* (YP_004136235.1), *Vibrio cholerae* (ZP_17776694.1), *Pseudomonas aeruginosa* (ZP_15628770.1), *Xylella fastidiosa* (NP_297336.1), *Methylocystis sp.* (YP_006593692.1), *Afipia sp.* (ZP_07027437.1), *Nitrosomonas sp.* (YP_004696048.1) and *Neisseria sp.* (ZP_06980365.1).

Table S1. X-ray crystallography data collection and refinement statistics

Values in parentheses are for the outer resolution shell.

| **Structure** | **RlmJ/CA** | | **RlmJ/GA*** | | **RlmJ/GA*A** |  |
| --- | --- | --- | --- | --- | --- | --- |
| PDB code | 7P9O |  | | 7P8Q | 7P9I |  |
| **Data collection** | | | | |  |  |
| Beamline | Soleil Promixa-1 (PX1) |  | | Soleil Promixa-2 (PX2) | Soleil Promixa-2 (PX2) |  |
| Wavelength | 0.978566 |  | | 0.978995 | 0.980112 |  |
| Space group | C2 |  | | C2 | P222 |  |
| Unit cell (a, b, c, α, β, γ) | 79.99 38.89 90.65  90 105.62 90 |  | | 80.32 38.3 90.8  90 105.36 90 | 62.48 57.59 79.44  90 98.14 90 |  |
| Resolution range (Å) | 39.37-2.09 (2.17-2.09) |  | | 39.5-2.29 (2.37-2.29) | 38.74-1.59 (1.65-1.59) |  |
| Total reflections | 106523 (9828) |  | | 74008 (7904) | 507816 (45837) |  |
| Unique reflections | 15973 (1492) |  | | 12282 (1108) | 74170 (6987) |  |
| Multiplicity | 6.7 (6.6) |  | | 6.0 (6.4) | 6.8 (6.6) |  |
| Completeness (%) | 99.13 (93.66) |  | | 98.80 (89.94) | 99.33 (94.28) |  |
| Mean I/σ(I) | 14.20 (3.00) |  | | 7.59 (2.45) | 15.47 (1.34) |  |
| R(merge) | 0.0829 (0.4230) |  | | 0.1765 (0.7592) | 0.0846 (1.338) |  |
| CC(1/2) | 0.998 (0.965) |  | | 0.989 (0.809) | 0.999 (0.584) |  |
| **Model composition** | | | | |  |  |
| Non-hydrogen atoms | 2331 |  | | 2334 | 5122 |  |
| Macromolecules | 2204 |  | | 2213 | 4369 |  |
| Solvent | 101 |  | | 50 | 567 |  |
| Protein residues | 274 |  | | 275 | 544 |  |
| **Refinement** | | | | |  |  |
| R(work) | 0.2472 (0.3041) |  | | 0.2253 (0.3869) | 0.1896 (0.3121) |  |
| R(free) | 0.3129 (0.3750) |  | | 0.2826 (0.5130) | 0.2149 (0.3424) |  |
| **Model validation** | | | | |  |  |
| All-atom clash score | 13.70 |  | | 5.77 | 3.70 |  |
| Rotamer outliers | 0.42 |  | | 0.42 | 0.22 |  |
| Average B-factor (Å^2^) | 22.93 |  | | 55.54 | 22.17 |  |
| *Ramachandran statistics (%):* | | |  | |  |  |
| Favoured (overall) | 94.81 |  | | 95.57 | 97.39 |  |
| Allowed (overall) | 5.19 |  | | 4.43 | 2.61 |  |
| Outliers (overall) | 0.00 |  | | 0.00 | 0.00 |  |
| *RMS deviations:* | | |  | |  |  |
| Bond length (Å) | 0.009 |  | | 0.013 | 0.008 |  |
| Bond angle (°) | 1.07 |  | | 1.44 | 0.90 |  |

**Supplementary Figure S39.** Bar chart showing the melting temperature (Tm in °C) for RlmJ (WT) and mutant proteins derived from the protein melting curves determined by differential scanning fluorimetry. The Tm of RlmJ and mutants as apo (no ligand) or in the presence of SAM are highlighted in light grey and dark grey respectively. Standard deviations are calculated from three analytical replicates.

1. X. F. Zhu, A. I. Scott *Synthetic Commun*. **2008**, *38*, 1346-1354.

   2 C. Atdjian, L. Iannazzo, E. Braud, M. Ethève-Quelquejeu *Eur. J. Org. Chem*. **2018**, 4411-4425. [↑](#footnote-ref-1)
2. [↑](#footnote-ref-2)
3. S. Bae, M. K. Lakshman *J. Am. Chem. Soc.* **2007**, *129*, 782-789. [↑](#footnote-ref-3)
4. [↑](#footnote-ref-4)
